# Immunogenic Nanovesicle‐Tandem‐Augmented Chemoimmunotherapy via Efficient Cancer‐Homing Delivery and Optimized Ordinal‐Interval Regime

**DOI:** 10.1002/advs.202205247

**Published:** 2022-12-01

**Authors:** Mengchi Sun, Wen Shi, Yuxia Wu, Zhonggui He, Jin Sun, Shuang Cai, Qiuhua Luo

**Affiliations:** ^1^ School of Pharmacy Shenyang Pharmaceutical University Shenyang Liaoning 110016 P. R. China; ^2^ Department of Pharmacy The First Hospital of China Medical University Shenyang Liaoning 110001 P. R. China; ^3^ Wuya College of Innovation Shenyang Pharmaceutical University Shenyang Liaoning 110016 P. R. China

**Keywords:** cancer‐homing delivery, drug delivery, ordinal‐interval regime, triple‐negative breast cancer, tumor‐derived extracellular vesicles

## Abstract

The strategy of combining immune checkpoint inhibitors (ICIs) with anthracycline is recommended by clinical guidelines for the standard‐of‐care treatment of triple‐negative breast cancer (TNBC). Nevertheless, several fundamental clinical principles are yet to be elucidated to achieve a great therapeutic effect, including cancer‐homing delivery efficiency and ordinal‐interval regime. Tumor‐derived extracellular vesicles (TDEVs), as vectors for intratumoral intercellular communication, can encapsulate therapeutic agents and home tumors. However, PD‐L1 overexpression in TDEVs leads to systemic immunosuppression during in vivo circulation, ultimately inhibiting intratumoral T activity. In this study, CRISPR/Cas9‐edited Pd‐l1^KO^ TDEV‐fusogenic anthracycline doxorubicin (DOX) liposomes with high drug encapsulation (97%) are fabricated, which homologously deliver DOX to breast cancer cells to intensify the immunogenic response and induce PD‐L1 overexpression in the tumor. By setting the stage for sensitizing tumors to ICIs, sequential treatment with disulfide‐linked PD1‐cross‐anchored TDEVs nanogels at one‐day interval could sustainably release PD1 in the tumor, triggering a high proportion of effector T cell‐mediated destruction of orthotopic and metastatic tumors without off‐target side effects in the 4T1‐bearing TNBC mouse model. Such a TDEV‐tandem‐augmented chemoimmunotherapeutic strategy with efficient cancer‐homing delivery capacity and optimized ordinal‐interval regime provides a solid foundation for developing chemoimmunotherapeutic formulations for TNBC therapy at the clinical level.

## Graphical abstract

Two key determinants included efficient cancer‐homing delivery and optimized ordinal‐interval regime for immunogenic nanovesicles‐tandem‐augmented chemoimmunotherapy. The tumor‐derived extracellular vesicles (TDEVs)‐tandem‐augmented chemoimmunotherapeutic strategy is utilized to prevent the immune surveillance caused by activating PD1/PD‐L1 pathway, proving to be effective in suppressing orthotopic tumors and minimizing pulmonary metastases.

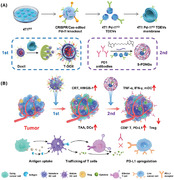

## 1 Introduction

Triple‐negative breast cancer (TNBC), characterized by high invasiveness and heterogeneity, is resistant to chemotherapy and targeted therapies, resulting in a median overall survival of only 8–13 months.^[^ ^1^, ^2^ ^]^ Although the clinical applications of immune checkpoint inhibitors (ICIs) have made initial progress in treating TNBC, a 5% objective response rate (ORR) makes it difficult for most patients to obtain a long‐term survival benefit.^[^ ^3^, ^4^ ^]^ Notably, PD‐L1‐positive patients with TNBC after the treatment with ICIs show higher ORR (19–23%), highlighting the importance of increasing PD1/PD‐L1 blockade sensitivity by upregulating PD‐L1 expression.^[^ ^5^, ^6^, ^7^ ^]^

Clinical treatment guidelines, such as the National Comprehensive Cancer Network (NCCN) guidelines, have recommended combined chemoimmunotherapy to improve the anticancer immune response for TNBC therapy.^[^ ^8^ ^]^ Anthracycline doxorubicin (DOX) is commonly used as a first‐line chemotherapeutic in combination with TNBC. Given previous clinical results, we found that PD‐L1 expression in tumor samples from patients with TNBC was markedly upregulated after treatment with DOX, which is of great value to patients with TNBC in enhancing the effects of immunotherapy.^[^ ^9^, ^10^, ^11^ ^]^ However, the ordinal‐interval regime for chemoimmunotherapy has not yet been systematically investigated. Moreover, there are other concerns regarding the limited intratumoral accumulation of DOX and its off‐target cardiotoxicity as well as immune‐related adverse effects (irAEs) caused by ICIs, all of which are key issues that require prompt solutions in clinical practice.^[^ ^12^, ^13^, ^14^, ^15^ ^]^ We expect that efficient cancer‐homing delivery capacity and optimized ordinal‐interval regime could further enhance the chemoimmunotherapeutic effect at the clinical level.

To verify our hypothesis, we proposed an innovative immunogenic nanovesicle‐tandem‐augmented strategy by leveraging tumor‐derived extracellular vesicles (TDEVs) to deliver chemoimmunotherapeutics. As is well known, TDEVs are recently emerging as promising vectors in delivering agents, including therapeutic genes, proteins, and small molecules, to tumors at orthotopic and metastatic sites based on their prominent tumor tropism property.^[^ ^16^, ^17^, ^18^ ^]^ In this study, we utilized the CRISPR/Cas9 gene‐edited bio‐engineered TDEVs from murine breast cancer cell (4T1) line with Pd‐l1 knockout (4T1 Pd‐l1^KO^) to prevent the immune surveillance caused by activated PD1/PD‐L1 pathway during an in vivo drug delivery process.^[^ ^19^, ^20^, ^21^, ^22^ ^]^ The obtained TDEV‐fusogenic anthracycline DOX liposomes, named “T‐DOX,” were constructed to achieve high encapsulation capacity and enrichment at tumor sites. Furthermore, T‐DOX‐mediated chemotherapy promoted significant upregulation of PD‐L1 expression with increasing levels of TNF‐*α* and IFN‐*γ*.

In light of this sensitized response of T‐DOX to ICIs, cancer‐homing disulfide‐linked PD1‐cross‐anchored TDEVs nanogel (S‐PDNG) was subsequently administered at one‐day intervals. The TDEV‐tandem‐augmented chemoimmunotherapeutic strategy with the indicated regimen was highly effective in suppressing orthotopic tumors and minimizing pulmonary metastases. Overall, the present study is the first to explore the simultaneous enhancement of cancer‐homing delivery capacity and ordinal‐interval regime for the optimal order and interval of administration to potentiate cancer immunotherapy. These results provide strong hints for designing multimodal TNBC therapies consisting of chemotherapeutics and ICIs in the clinic.

## 2 Results and Discussion

### 2.1 Triple‐Negative Breast Cancer Patient Sample Analysis

**Figure 1:**
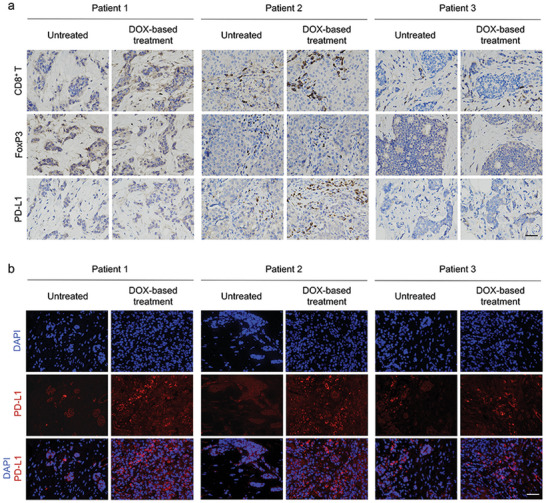
Tumor sample analysis from patients with TNBC. a) Representative immunohistochemistry (IHC) images analyzing CD8^+^, Foxp3^+^, and PD‐L1 of tumor tissues from patients with TNBC. Scale bar = 50 µm. Untreated represents samples from patients without any therapy. DOX‐based treatment represents tumor samples from patients after four courses of neoadjuvant chemotherapy consisting of a DOX‐containing regimen. b) Representative immunofluorescence (IF) images of tumor tissues using DAPI (blue) and PD‐L1 (red). Scale bar = 50 µm.

We collected clinical tumor samples from patients with TNBC before and after four courses of AC (DOX plus cyclophosphamide) treatment for preoperative neoadjuvant therapy. The biopsy sections were examined by immunohistochemistry (IHC) and immunofluorescence (IF) staining to detect CD8^+^ T cells, Tregs, and PD‐L1‐positive cells. As illustrated in **Figure** **1**a, we noticed profound CD8^+^ T cell infiltration after the induction of anthracycline DOX treatment, in parallel with decreased Foxp3^+^ Tregs. Moreover, compared with untreated patients, we observed a trend toward increased PD‐L1 expression in TNBC biopsy sections from patients receiving AC treatment. Similarly, according to the IF staining images, intense fluorescence signals were observed in the tumors after DOX treatment, revealing the upregulation of PD‐L1 expression (Figure 1b). The results indicated that DOX played a significant role in reversing the immunosuppressive tumor microenvironment (TME) and promoting PD‐L1 expression, thus supporting further investigation of the combination of ICIs with DOX‐based chemotherapy for TNBC treatment. We speculate that upregulated PD‐L1 expression and an immune‐active TME could be particularly conducive to the subsequent targeted anti‐PD1 therapy at an early stage to achieve maximum synergistic efficacy.

### 2.2 Design and Characterization of Tumor‐Derived Extracellular Vesicles Pd‐l1^KO^‐Fusogenic Doxorubicin Liposomes (T‐DOX)

**Figure 2:**
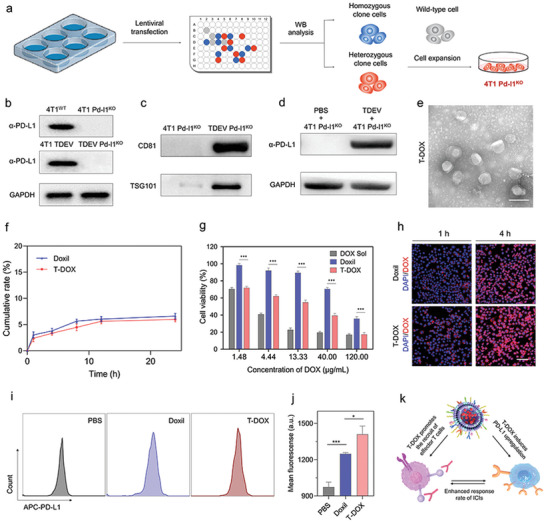
In vitro characteristics of T‐DOX. a) Schematic representation of 4T1 Pd‐l1^KO^ using CRISPR/Cas9 gene‐editing technique. b) Western blot assay of PD‐L1 knockout validation for characteristic expression of PD‐L1, 4T1^WT^ cells as the control. c) Detection of TSG101 and CD81 proteins for 4T1 Pd‐l1^KO^ derived TDEVs. d) 4T1^WT^ cell‐derived TDEVs or 4T1 Pd‐l1^KO^ cell‐derived TDEVs were added to 4T1 Pd‐l1^KO^ cells. After 24 h incubation, cells were washed with PBS four times, lysed in lysis buffer, and subjected to western blotting using the indicated antibodies. e) TEM image of T‐DOX. Scale bar = 200 nm. f) In vitro release profile of Doxil and T‐DOX in PBS (pH 7.4). g) Cytotoxicity of DOX Sol, Doxil, and T‐DOX against 4T1 cells for 24 h. h) CLSM imaging of 4T1 cells after incubating with Doxil or T‐DOX for 1 and 4 h. Scale bar = 50 µm. i,j) Flow cytometry analysis of PD‐L1‐positive cells in 4T1 cells treated with various treatments. k) Schematic representation of the PD1 inhibitor and upregulated PD‐L1 interaction in tumor cells. Significant values are presented as mean ± SD (*n* = 3); *p < 0.05; **p < 0.01; ***p < 0.001.

Previous studies have shown that tumor cells secrete abundant PD‐L1‐bearing TDEVs that circulate in the tumor, profoundly inhibit T cell function, and promote immune tolerance.^[^ ^20^, ^22^ ^]^ Therefore, we used CRISPR/Cas9 gene‐editing technique to knockout the PD‐L1 gene in 4T1 cells (4T1 Pd‐l1^KO^, **Figure** **2**a). We used western blotting to verify PD‐L1 expression in 4T1 Pd‐l1^KO^ cells and released TDEVs. As illustrated in Figure 2b, we found that wild‐type 4T1 cells (4T1^WT^ cells) exhibited PD‐L1 expression, similar to the secreted TDEVs (4T1 TDEVs). In comparison, 4T1 Pd‐l1^KO^ cells did not express PD‐L1‐related proteins or did the derived TDEVs. In a subsequent study, 4T1 Pd‐l1^KO^ cell‐derived TDEVs were collected using serial extrusion and stepwise centrifugation (Figure S1, Supporting Information) and were chosen as endogenous vectors. The transmission electron microscopy (TEM) images of the TDEVs indicated a typical saucer‐shaped structure with an average size of ≈100 nm (Figure S2, Supporting Information). Meanwhile, western blot analysis revealed high expression of the characteristic TDEVs’ markers TSG101 and CD81 (Figure 2c), demonstrating the successful isolation of 4T1 Pd‐l1^KO^ cell‐derived TDEVs.^[^ ^23^ ^]^ We detected the transfer of PD‐L1 from 4T1^WT^ cell‐derived TDEVs to 4T1 Pd‐1l^KO^ cells, suggesting that TDEVs could transfer functional PD‐L1 to other tumor cells (Figure 2d). These results also confirmed the importance of knocking out PD‐L1 from the delivery system.

Next, we evaluated the pharmacological properties of 4T1 Pd‐l1^KO^ cell‐derived TDEVs. First, we sought to determine the experimental conditions that allowed optimal co‐fusion of TDEVs and liposomes. TDEVs were labeled with the green membrane dye DiO (TDEVs^DiO^) and liposomes with the red membrane dye DiL (Lip^DiL^).^[^ ^24^ ^]^ Co‐localized yellow fluorescent signals from DiO and DiL were observed during the fusion of T‐DOX using confocal laser scanning microscopy (CLSM). Figure S4, Supporting Information, shows that TDEVs^DiO^ and Lip^DiL^ were scattered separately after a simple physical mixture. Through repeated co‐extrusion by a 220 nm polycarbonate porous membrane on an extruder, the red and green fluorescence signals exhibited a complete overlap of the yellow‐light spots The obtained T‐DOX displayed liposomal morphological characteristics of a spherical vesicular structure in TEM images, with an average size of ≈120 nm (Figure 2e). Moreover, atomic force microscopy (AFM) showed that T‐DOX was also characterized by a rougher surface morphology, in contrast to the surface of Doxil, suggesting the convergence between TDEVs and liposomes (Figure S3, Supporting Information). These results demonstrate the complete fusion and integrity of the membranes associated with T‐DOX.

The encapsulation efficiency (EE) of T‐DOX first increased and then decreased with increasing TDEVs’ content. When the lipid: TDEVs protein ratio was 1:0.2, the encapsulation rate of T‐DOX reached a maximum of 97% (Figure S5, Supporting Information). This is likely because the increased amount of protein within T‐DOX occupied the DOX hosting site, resulting in reduced encapsulation of DOX.

Both Doxil and T‐DOX demonstrated a similar and sustained release profile, displaying ≈7% DOX release under physiological conditions (pH 7.4, PBS) (Figure 2f). T‐DOX showed desirable colloidal stability after incubation in PBS (pH 7.4) containing 10% fetal bovine serum (FBS) for 30 h (Figure S6, Supporting Information). These results indicate that the fusion of TDEVs and liposomes did not alter the release profile or stability of Doxil.

### 2.3 In Vitro Cellular Uptake and Cytotoxicity

To investigate whether T‐DOX retained the cancer‐homing ability of TDEVs, we performed a cellular uptake study using 4T1 cells, a widely used murine model of TNBC.^[^ ^25^ ^]^ As shown in Figure 2h, we found significantly higher fluorescence intensity in 4T1 cells incubated with T‐DOX at predetermined time points of 1 and 4 h. Quantitative detection results using flow cytometry were consistent with the CLSM results, demonstrating 1.5–1.7 times higher fluorescence intensity of T‐DOX than Doxil at 4, 6, and 8 h (Figure S7, Supporting Information). In vitro cytotoxicity was investigated using the MTT assay. We first performed an in vitro evaluation of the toxicity of the blank vector. As shown from the Figure S8, Supporting Information, we did not see an obvious effect on the level of cytotoxic activity against 4T1 cells in the presence of blank vector ranging from 10 to 160 µg mL^−1^ at 48 h. Second, we observed that T‐DOX (IC50:12.98 µg mL^−1^) demonstrated remarkably lower cell viability than Doxil (IC50:75.98 µg mL^−1^) at 24 h (Figure 2g). A good agreement was observed at 48 h (Figure S9, Supporting Information, IC50:26.21 µg mL^−1^ for Doxil and 1.98 µg mL^−1^ for T‐DOX). The enhanced cellular uptake and cytotoxicity of T‐DOX may result from homologous membrane‐associated proteins on TDEVs, making it a favorable delivery vector for targeting and precise cancer therapy.

### 2.4 PD‐L1 Induction Assay

Previous studies have confirmed that several chemotherapeutics (such as DOX, carboplatin, gemcitabine, and paclitaxel) can induce enrichment of CD47^+^/CD73^+^/PDL1^+^ TNBC cells.^[^ ^9^, ^26^ ^]^ We also questioned whether the T‐DOX had a significant impact on PD‐L1 expression. After incubation with various treatment groups for 24 h, the cells were collected and stained with fluorescent antigen‐presenting cell (APC) anti‐PD‐L1 to detect PD‐L1‐positive cells using flow cytometry. As indicated in Figure 2i, the T‐DOX group produced a more significant number of PD‐L1‐positive cells than the Doxil. According to the quantitative analysis, after a 24 h induction, T‐DOX (0.5 µg mL^−1^) significantly induced PD‐L1 expression in comparison with Doxil (Figure 2j). We speculated that this might be due to enhanced internalization of T‐DOX by 4T1 cells. This was consistent with the phenomenon observed in clinical practice and thus laid the foundation for sensitizing subsequent ICI therapy (Figure 2k).

### 2.5 Immunostimulatory Activity of T‐DOX

To evaluate the immunostimulatory activity of T‐DOX, we first employed sodium dodecyl sulfate‐polyacrylamide gel electrophoresis (SDS‐PAGE) to compare tumor‐associated antigens on 4T1 cells, TDEVs, and T‐DOX.^[^ ^27^ ^]^ The protein profiles of TDEVs and T‐DOX displayed a high degree of consistent similarity, indicating good preservation of proteins distinctively inherited from 4T1 cells (Figure S10, Supporting Information). The retained protein could be necessary for accomplishing relevant biological functions and cancer‐homing targeting as a delivery carrier.

**Figure 3:**
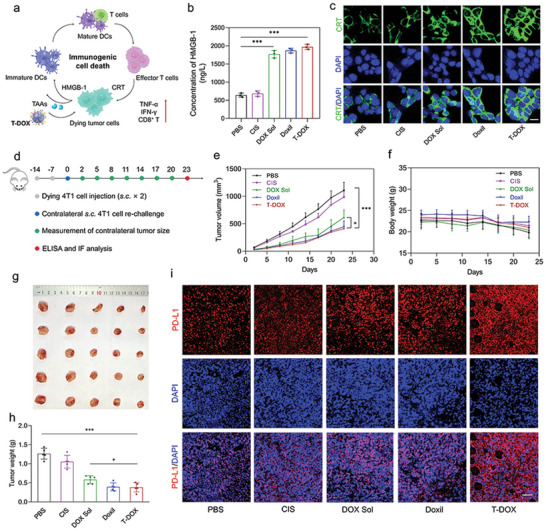
DOX‐induced ICD provided a successful anti‐TNBC vaccination approach and favorable TME that facilitated ICI therapy. a) Schematic to explain the effect of T‐DOX on promoting ICD and inducing PD‐L1 upregulation in tumors. b) HMGB‐1 release and c) CRT exposure in 4T1 cells after incubation with PBS, cis‐platinum (CIS), DOX Sol, Doxil, and T‐DOX for 24 h (*n* = 3). Scale bar = 10 µm. d) Animal experimentation used two vaccination rounds one week apart, followed by injecting live 4T1 cells subcutaneously (s.c.) on the contralateral side. e) 4T1 tumor growth curves in the contralateral flank. f) Body weight of tumor‐bearing mice. g) Representative tumor images and h) tumor weight from each group after euthanizing the animal on day 23 (*n* = 5). i) PD‐L1 staining of tumor slices after various treatments. Scale bar = 50 µm. Significant values are presented as mean ±SD; *p < 0.05; **p < 0.01; ***p < 0.001.

Immunogenic cell death (ICD) converts dying cancer cells into a therapeutic vaccine accompanied by damage‐associated molecular patterns, initiating a tumor antigen‐specific immune response.^[^ ^28^, ^29^, ^30^ ^]^ The anthracyclines hold the potential to induce an ICD effect in several cancer cell lines. As a classic anthracycline, DOX not only directly induces tumor cell apoptosis through DNA damage but also promotes antitumor immunity by stimulating ICD. DOX enhanced the immunogenicity of TNBC cells and stimulated DC maturation (**Figure** **3**a).^[^ ^31^ ^]^ Using calreticulin (CRT) exposure and high mobility group box 1 protein (HMGB‐1) release as surrogate markers for drug‐induced tumor cell immunogenicity, we found that all DOX‐containing formulations, including DOX solution (DOX Sol), Doxil, and T‐DOX, induced immunogenic effects on 4T1 cells by increasing HMGB1 release and CRT exposure on the cell membrane (Figure 3b,c).^[^ ^32^ ^]^ To identify the host immune response, we used a vaccination approach to confirm whether the above treatments could stimulate adaptive and innate anti‐TNBC immunities in vivo. BALB/c mice were subcutaneously injected with 4T1 cells pre‐incubated with various treatments on two occasions.^[^ ^33^ ^]^ Seven days later, the mice were rechallenged with live 4T1 cells on the contralateral side (Figure 3d). The vaccination of DOX Sol‐, Doxil‐, and T‐DOX‐treated cells significantly suppressed tumor growth on the contralateral side, indicating a solid systemic immune response against the re‐challenged tumors.

In contrast, CIS treatment had little effect on tumor growth (Figure 3e,g,h). All treatments showed a good safety profile (Figure 3f). However, T‐DOX triggered an enormous amount of IFN‐*γ* and TNF‐*α* release, reflecting a potent systemic immune response (Figure S11, Supporting Information). Additionally, tumor tissues showed more intense green fluorescence, revealing that the in vivo CRT exposure was remarkably increased after T‐DOX treatment (Figure S12, Supporting Information). It is also worth noting that T‐DOX could upregulate PD‐L1 expression in tumor tissues in response to ICD (Figure 3i), elucidating the necessity and suitability of combining PD1 blockers in TNBC. These results demonstrated that T‐DOX could effectively create a “re‐educated” immunogenic TME with enhanced PD‐L1 expression by amplifying the ICD effect of DOX, which may ultimately facilitate subsequent ICI therapy.

### 2.6 In Vivo Cancer‐Homing Verification

The in vivo cancer‐homing capability of T‐DOX was determined using an orthotopic 4T1 tumor‐bearing mouse model. BALB/c mice were treated with DiR‐labeled Doxil (Doxil^DiR^) or DiR‐labeled T‐DOX (T‐DOX^DiR^), and imaged using the in vivo imaging system (IVIS) Lumina XR system at predetermined time intervals. As indicated in Figure S13, Supporting Information, the fluorescence intensities of Doxil^DiR^ and T‐DOX^DiR^ peaked at the tumor site between 12 and 36 h after intravenous administration. Moreover, compared to Doxil^DiR^, T‐DOX^DiR^ showed significantly higher fluorescence intensity in tumor tissues over time. The enhanced tumor accumulation of T‐DOX^DiR^ may be closely related to homologous homing and the high penetration ability associated with TDEVs, as indicated by the cell uptake study.

### 2.7 Characteristic and In Vivo Antitumor Study of Tumor Microenvironment‐Responsive PD1‐Cross‐Anchored Tumor‐Derived Extracellular Vesicles Nanogels (S‐PDNGs)

**Figure 4:**
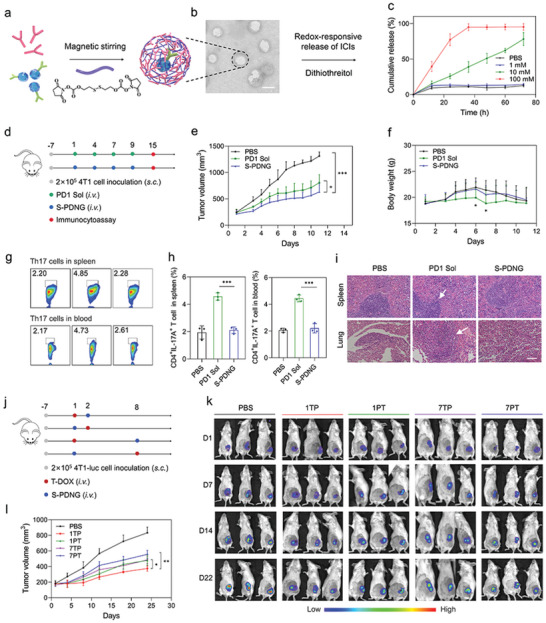
Characterization of TME‐responsive S‐PDNGs and optimized sequential dosing of combined chemoimmunotherapy. a) Schematic to explain the fabrication of S‐PDNGs and redox‐responsive release in the TME. b) TEM image of S‐PDNGs. Scale bar = 100 nm. c) In vitro drug release profiles of PD1 from S‐PDNGs in PBS (pH 7.4) containing various concentrations of DTT (*n* = 3). d) Orthotopic 4T1 tumor‐bearing mice (*n* = 5) were intravenously(i.v.) injected with PD1 Sol or S‐PDNGs (50 µg PD1/mouse) for a total of four administrations. e) 4T1 tumor growth curve and f) body weight of mice treated with various treatments. g) Representative flow cytometry plots and h) quantitative analysis of Th17 cells in spleen and blood (*n* = 3). i) H&E staining of the spleens and lungs harvested on day 24 from tumor‐bearing mice treated with indicated formulations. j) Orthotopic 4T1‐luc tumor‐bearing mice (*n* = 3) were i.v. injected with combined S‐PDNGs (50 µg PD1/mouse) and T‐DOX (5 mg kg^−1^) for a 1‐day or 7‐day interval following indicated orders. k) In vivo bioluminescence images to track the tumor growth in the different groups of mice after various treatments. l) 4T1 tumor growth curve following sequential dosing of T‐DOX and S‐PDNGs. Significant values are presented as mean ± SD; *p < 0.05; **p < 0.01; ***p < 0.001.

Since systemically applied ICIs may disrupt the balance in maintaining immunological homeostasis and lead to autoimmune‐like side effects, we designed TME‐responsive disulfide‐linked PD1‐cross‐anchored TDEVs nanogels (S‐PDNGs) to increase the cancer‐homing ability of ICIs and reduce systemic irAE (**Figure** **4**a). Our previous study confirmed the redox responsiveness of the disulfide bonds. This provides a good foundation for developing a drug delivery systemDDS for cancer therapy.^[^ ^34^, ^35^, ^36^ ^]^ Therefore, we first prepared a disulfide bond‐bridged PD1 nano‐cluster (S‐PD), in which the TEM showed a distinct spherical structure with a particle size of ≈150 nm (Figure S14, Supporting Information). Then, the PD1 solution was co‐incubated with TDEVs with disulfide bond‐containing linkers in a homothermal shaker at 37 °C for 1 h to produce S‐PDNGs with cancer homing and reduction sensitivity. As indicated in Figure 4b, the TEM image shows a slight increase in the particle size of the TDEVs (≈200 nm), confirming the successful assembly of PD1 and TDEVs into one system. A reduction‐responsiveness assay of S‐PDNGs was conducted using dithiothreitol (DTT) as a reductant simulator.^[^ ^37^ ^]^ The in vitro release profile showed that S‐PDNGs could hardly release PD1 inhibitors in blank release medium without DTT. In contrast, it offered a pronounced release of PD1 inhibitor by 25% and 75% within 24 h in the medium containing 10 mm DTT and 100 mm DTT, respectively (Figure 4c), indicating that the PD1 inhibitor could be released quickly and precisely into the tumor redox microenvironment.

Furthermore, we examined the in vivo efficacy of the two different PD1‐based formulations in orthotopic 4T1 tumor‐bearing mice. The S‐PDNGs group showed significant tumor regression against 4T1 tumors and lower toxicity than the PD1 solution (PD1 Sol) group (Figure 4e,f). After the pharmacodynamic experiment, we collected tumors and spleens on day 15 to evaluate CD8^+^ T cell infiltration. According to the flow cytometry study, S‐PDNGs led to a high production of CD8^+^ T cells in tumors and splenocytes (Figure S15a, Supporting Information). Brighter green fluorescence was observed in anti‐CD8‐FITC‐stained tumor slices, demonstrating that a large number of CD8^+^ T cells infiltrated the tumor tissue (Figure S16, Supporting Information). Compared to the lung tissues collected from treated mice, we found more metastatic nodules in mice treated with PD1 Sol (Figure S15b, Supporting Information).

Moreover, we noticed that PD1 Sol‐treated mice showed significant weight loss, and two mice died on days 6 and 7, respectively (Figure 4f). In contrast, mice treated with S‐PDNGs remained stable compared to those treated with PBS, demonstrating an excellent safety profile. Previous studies have confirmed that an imbalance between Th17 cells and Tregs plays a crucial role in breaking immune tolerance.^[^ ^38^, ^39^ ^]^ IL‐17, secreted by Th17 cells, could induce an inflammatory reaction significantly correlated with an increased risk of irAEs. Thus, the Th17 cell ratio can be used to monitor irAEs associated with ICIs. Compared to PBS and S‐PDNGs, treatment with PD1 Sol generated an increased quantity of Th17 cells in the spleen and peripheral blood of the mice (Figure 4g,h). Hematoxylin and eosin (H&E) staining of spleen and lung slices indicated an increased number of inflammatory cells in the PD1 Sol‐treated group, reflecting the potential risk of irAEs when systematically injected with PD1 Sol (Figure 4i). In contrast, there was no significant difference between the S‐PDNG and PBS groups. The above data indicate that S‐PDNGs, endowed with cancer‐homing and responsiveness, could significantly improve the in vivo therapeutic efficacy and reduce the severe toxicity of ICIs due to the induction of CD8^+^ T cells and reduction of Th 17 cells.

### 2.8 Optimized Strategy of T‐DOX and S‐PDNGs

Based on current clinical practice, we hypothesized that a combination strategy of T‐DOX and S‐PDNGs may contribute to a remarkable synergistic anticancer chemoimmunotherapeutic effect. To maximize the efficacy of chemoimmunotherapy, we aimed to clarify the ordinal‐interval regimen for chemoimmunotherapeutics. We performed an in vivo bioluminescence study to visually monitor tumor growth in 4T1‐luc tumor‐bearing mice sequentially treated with T‐DOX and S‐PDNGs at 1‐day and 7‐day intervals, respectively (Figure 4j). From the in vivo bioluminescence images on day 22, we found that mice administered PBS showed distinct tumor growth. Among the sequential treatment groups, the 1‐day interval of T‐DOX followed by S‐PDNGs showed the optimal tumor‐suppressive effect, indicated by the weakest bioluminescence, which is also in accordance with the tumor growth curves recorded using a vernier caliper (Figure 4k,l). It is likely that T‐DOX could kill a large number of cancer cells efficiently via increased cell uptake and induce ICD, releasing “eat me” signals to facilitate CD8^+^ T cell infiltration into the tumor. Furthermore, our analyses of sequential on‐treatment immunomodulatory cytokines revealed that the 1‐day interval regime in the indicated order was associated with a significantly higher IFN‐*γ* and TNF‐*α* release in tumor tissues than in other treatment regimes (Figure S17, Supporting Information). The body weights of the mice remained stable without severe weight loss among the different treatment groups during the experiment (Figure S18, Supporting Information). These results led us to the following conclusions: 1) The 1‐day interval regime appeared to be superior to the 7‐day interval in retarding tumor growth with the release of higher amounts of IFN‐*γ* and TNF‐*α*; 2) T‐DOX followed by S‐PDNGs offered more advantages over the opposite sequence, regardless of the time interval. Based on previous results, we speculate that the high immunogenicity and upregulated PD‐L1 expression in tumors induced by T‐DOX were expected to be particularly conducive to the subsequent targeted anti‐PD1 therapy at an early stage to achieve maximum synergistic efficacy. In this way, optimizing the sequence and interval of combination therapy can synergistically enhance TNBC immunotherapy.

### 2.9 In Vivo Synergistic Antitumor Effect on the Orthotopic 4T1 Tumor Model

**Figure 5:**
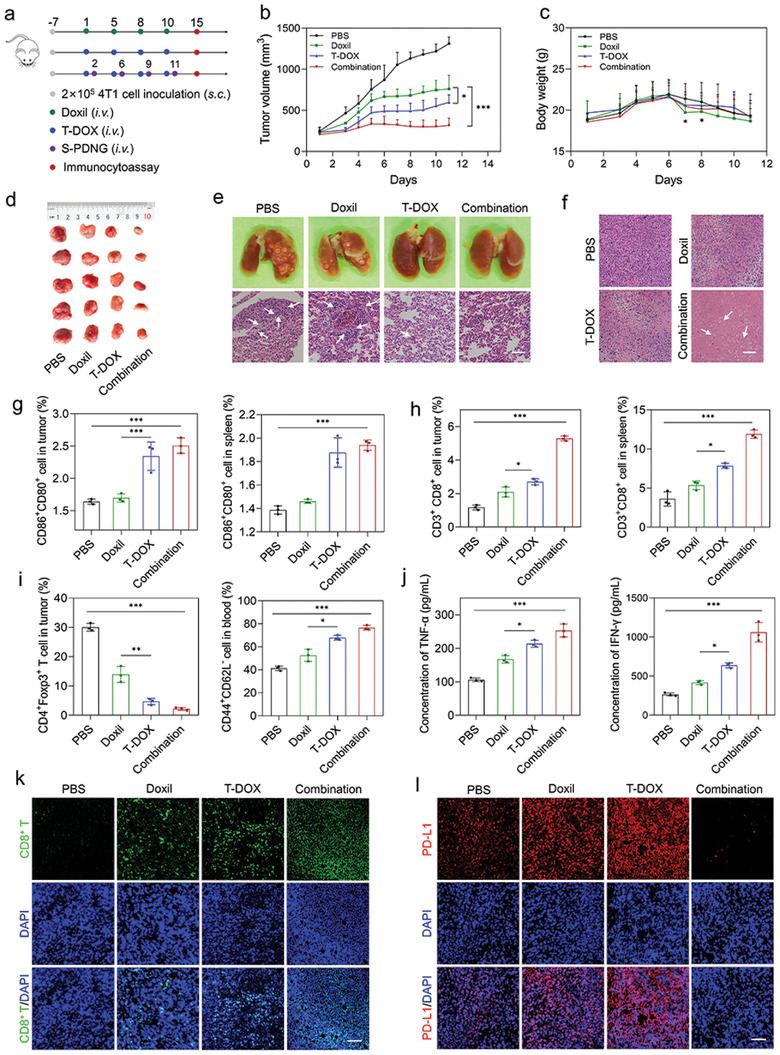
Chemoimmunotherapy for induction of T cell responses and elimination of 4T1 tumors. a) Orthotopic 4T1 tumor‐bearing mice (*n* = 5) were i.v. injected with PBS, Doxil, T‐DOX, and combination therapy (T‐DOX plus S‐PDNGs) for a total of four administrations. b) 4T1 tumor growth curve and c) body weight of mice treated with various treatments. d) Representative tumor images from each group after euthanizing the animal on day 11. e) Lung tissues and H&E staining of lung slices collected from various treatments. Red circles demonstrate the visible metastatic site. White arrows demonstrate the lung metastases. Scale bar = 100 µm. f) H&E staining of tumor slices collected from various treatments. White arrows demonstrate the apoptosis and necrosis cells. Scale bar = 100 µm. g) DC ratios and h) CD8^+^ T cell in tumors and splenocytes of the 4T1 tumor‐bearing mice after various treatments (*n* = 3). i) Treg and memory T cell ratios in tumors and blood of the 4T1 tumor‐bearing mice after various treatments (*n* = 3). j) Analysis of TNF‐*α* and IFN‐*γ* in tumors after various treatments (*n* = 3). k) CD8 and l) PD‐L1 staining of tumor slices after various treatments. Scale bar = 50 µm. Significant values are presented as mean ± SD; *p < 0.05; **p < 0.01; ***p < 0.001.

Having confirmed the optimized regime of the combined chemoimmunotherapy, we questioned whether the combination therapy of T‐DOX plus S‐PDNGs could exert the best synergistic therapeutic efficacy against orthotopic 4T1 tumors (**Figure** **5**a). The results from in vivo pharmacodynamic experiments showed that the combination group produced an impressive tumor inhibition rate of 76%, which was much higher than that of T‐DOX (54%) and Doxil (42%) (Figure 5b,d). There was no significant difference in body weight between the treatment groups, confirming the safety of the therapeutic regimens (Figure 5c). In addition, we found that the PBS‐ and DOX‐treated groups exhibited severe lung metastases. In contrast, T‐DOX showed a potent inhibitory effect on metastatic tumors to some extent. Mice receiving T‐DOX plus S‐PDNGs showed no evident metastatic pulmonary nodules and the most extensive cell dissolution, with a large area of tumor apoptosis and necrosis (Figure 5e,f).

### 2.10 Mechanism Analysis of Combining T‐DOX with S‐PDNGs for Immunochemotherapy

To explore the immunoregulatory effects of the combination therapy, we analyzed the proportion of various immune cells. Figure 5g shows that T‐DOX and combination therapy significantly promoted the maturation of DCs in the tumors and spleens. This may be due to the superior cancer‐homing ability of T‐DOX, which resulted in an increased accumulation of DOX at tumor sites that triggered augmented ICD‐associated antigen exposure, capture, and presentation for DC recruitment. The maturation of DCs is a prerequisite for initiating innate and adaptive immune responses.^[^ ^40^ ^]^

Mature DCs, as typical APCs, would present surface antigens to T cells and subsequently stimulate the activation of T cells.^[^ ^41^, ^42^ ^]^ We then evaluated the proportion of CD8^+^ T cells in tumors and spleens. As shown in Figure 5h, the combination therapy facilitated the highest infiltration of CD3^+^CD8^+^ T cells in tumors and splenocytes with a 2.1‐ and 2.3‐fold increase compared to Doxil, respectively, demonstrating potent systemic immune activation. Qualitative assessment using CLSM also revealed a boosted fluorescence intensity in tumor slices after the combination therapy, in agreement with the flow cytometry analysis (Figure 5k). In addition, tumors showed a prominent increase in TNF‐*α* and IFN‐*γ* levels compared to the other groups, encouraging a solid antitumor immune response (Figure 5j).

Tregs constitute an essential part of the immunosuppressive TME that restrains antitumor immunity.^[^ ^43^, ^44^, ^45^ ^]^ We found that T‐DOX and combination therapy significantly decreased the proportion of CD4^+^Foxp3^+^ T cells in tumors and splenocytes (Figure 5i and Figure S19a, Supporting Information), suggesting that these two treatments could effectively reverse the immunosuppressive TME.^[^ ^46^ ^]^ Additionally, a profound proportion of memory T cells, indicated by CD44^+^CD62L^−^ cells, was found in peripheral blood and splenocytes induced by T‐DOX and combination therapy, which is of far‐reaching significance for long‐term tumor immunization and may account for the potential establishment of durable immunity with a robust inhibitory effect on lung metastases and prevent the recurrence of TNBC (Figure 5i and Figure S19b, Supporting Information).^[^ ^47^, ^48^ ^]^

In addition, based on previous results, we indicated that T‐DOX could promote PD‐L1 expression in tumor cells in vitro. Therefore, we investigated whether T‐DOX could induce the upregulation of PD‐L1 expression in vivo. We examined PD‐L1 expression in tumor slices treated with various treatments using IF staining. The induction of T‐DOX significantly upregulated PD‐L1 expression compared to Doxil, which is in accordance with in vitro results of PD‐L1 induction. Notably, T‐DOX plus S‐PDNGs resulted in an outstanding downregulation of PD‐L1 expression, indicating a comparatively good prognosis for TNBC (Figure 5l). Since the binding of PD1 and its ligands (PD‐L1) is considered the critical inhibitory pathway in T cell proliferation, a further blockade of PD1 is highly recommended to restore the activity of effector T cells for continuous immune responses according to clinical guidelines.

As anticipated, the delivery of DOX via a TDEV‐based biohybrid system could augment its immunostimulatory effect by recruiting a substantial number of DCs and activating the highest proportion of functional CTLs, in parallel with Treg reduction. More importantly, treatment with T‐DOX led to a significant increase in PD‐L1 expression in tumors, which paved the way for improving the ORR and therapeutic effect of subsequent ICI treatment. Therefore, by coordinating the sequential administration of T‐DOX and S‐PDNGs, combined chemoimmunotherapy offers a synergistic effect on alleviating the low immunogenicity associated with TNBC and improving its low response rate to immunotherapy.

### 2.11 Safety Evaluation

For safety evaluation, we performed pathology analysis using H&E staining of the major organs. As shown in Figure S20, Supporting Information, we did not observe any abnormalities in the histological structure of the heart, liver, spleen, and lung from Doxil, T‐DOX, or combination treatment. The hepatorenal and renal functions of mice in all groups remained normal (Figure S21, Supporting Information). These results strongly indicate that combined chemoimmunotherapy with T‐DOX and S‐PDNGs is an effective and safe strategy for the treatment of TNBC.

## 3 Conclusions

In summary, we demonstrated that an efficient CRISPR/Cas9‐edited TDEV‐based biohybrid system for DOX delivery could be used for a synergistic immunotherapy response based on the induction of ICD and PD‐L1 upregulation in TNBC. An optimized ordinal‐interval regimen of S‐PDNGs could further aid in reversing the immunosuppressive microenvironment and increasing the effectiveness of cancer immunotherapy. Since TNBC is resistant to most treatment modalities, well‐designed chemoimmunotherapy would greatly benefit patients with TNBC in the clinic owing to its high efficiency and low toxicity. This study also offers a profound exploration of sensitizing ICIs and potentiating cancer immunotherapy by finely adjusting the order and interval of administration. The translational potential of this study may extend beyond TNBC to other malignant “cold” tumors associated with an immunosuppressive microenvironment.

## 4 Experimental Section

### Triple‐Negative Breast Cancer Patient Samples

TNBC tissue specimens were obtained from the Department of Breast Surgery of the First Hospital of China Medical University and approved by the guidelines approved by the Institutional Review Board of the First Clinical Hospital of China Medical University. Written informed consent was obtained from all patients. All patients with TNBC endured four courses of neoadjuvant chemotherapy consisting of a DOX‐containing regimen. CD8, Foxp3, and PD‐L1 expression in tumor tissues was examined using IHC. Immunofluorescence (IF) staining was performed to determine the fluorescence intensity of PD‐L1.

### Cell Lines and Animals

4T1 and 4T1 Pd‐l1^KO^ cells were cultured in RPIM 1640 supplemented with 10% FBS, 100 U mL^−1^ penicillin, and 100 µg mL^−1^ streptomycin at 37 °C under 5% CO_2_. 4T1 cells were subcutaneously inoculated into the left mammary fat pad of female BALB/c mice to establish an orthotopic tumor model. For bioluminescence imaging, 4T1 cells were stably transfected with luciferase‐expressing lentivirus (4T1‐luc). All experiments were performed in accordance with guidelines approved by the Institutional Animal Ethical Care Committee (IAEC) of China Medical University.

### Generation of 4T1 Pd‐l1^KO^ Cell Line

The lentiCRISPR v2 plasmid encoding sgRNA and Cas9 was obtained from Addgene (#52 961). Sequences of sgRNAs targeting mouse PD‐L1 were designed using the online CRISPR design developed by Dr. Zhang Feng's laboratory (http://zlab.bio/guide‐design‐resources). Pairs of sgRNA oligos for each targeting site were annealed and ligated into the BsmBI restriction site (New England Biolabs), linearizing lentiCRISPR v2.

### Isolation of 4T1 Pd‐l1^KO^ Cell‐Derived Exosomes (Tumor‐Derived Extracellular Vesicles)

4T1 Pd‐l1^KO^ cells were grown under 5% CO_2_ at 37 °C for 24 h until they reached 80% confluence. Afterward, the culture medium was replaced with serum‐free medium for another 48 h, after which the supernatant was collected for use. TDEVs were separated by differential centrifugation using a previously described method: the collected supernatant was centrifuged at 4 °C for 10 min at 300 × *g*, then at 2000 × *g* for 10 min, and at 10 000 × *g* for 30 min, followed by ultracentrifugation at 4 °C at 100 000 × *g* for 90 min. Exosomes were resuspended in a hypotonic buffer containing a protease inhibitor cocktail and stored at 4 °C for 12 h. The mixture was ultracentrifuged at 100 000 × *g* for 5 h to obtain TDEVs. The morphology of the TDEVs was observed using TEM (JEM‐2100, JEOL Ltd., Japan). An aliquot of 10 the sample was deposited on a copper grid and stained with phosphotungstic acid. The dried grids were examined using a transmission electron microscope at 80 kV. Western blot analysis was performed to identify exosome markers.

### Intercellular Transfer of Exosomal PD‐L1

Exosomes derived from wild‐type 4T1 cells (4T1 TDEVs) or PBS were added to the 4T1 Pd‐l1^KO^ cells. Following 24‐h incubation, the cells were washed with PBS three times, lysed in lysis buffer, and subjected to western blotting using the PD‐L1 antibody. GAPDH was used as an internal standard.

### Preparation and Characterization of T‐DOX

T‐DOX was prepared using a membrane extrusion method. Briefly, Doxil (containing 1 mg of lipids) and TDEVs (containing 0.2 mg of proteins) were mixed to a final volume of 0.5 mL by vortexing and sonicating for 30 s pulses on/off for 2 min, and then co‐extruded with 400 and 200 nm polycarbonate membrane filters 10 times (Antos Nanotechnology Co., Ltd). The obtained T‐DOX was diluted with a suitable volume of PBS, deposited on a copper grid, and stained with phosphotungstic acid. The dried grids were examined under a transmission electron microscope at 80 kV. AFM was used to determine the surface morphologies of the particles. Dynamic light scattering was used to determine the size distribution and polydispersity index of T‐DOX.

### PD‐L1 Induction Assay

4T1 cells (1.5 × 10^5^) were seeded in 24‐well plates overnight. Cells were then incubated with PBS, Doxil and T‐DOX (0.5 µg mL^−1^) for 24 h. The medium supernatant was removed by centrifugation at 1000 × *g* for 3 min. The harvested cells were washed twice and then resuspended in 100 µL of cell staining buffer (BioLegend, Cat.420201). The cells were incubated with APC anti‐mouse PD‐L1 antibody for 30 min on ice and then resuspended in 500 µL cell staining buffer. PD‐L1‐positive cells were detected by flow cytometry (BD, FACS AriaIII, USA). Fluorescence of the PD‐L1 antibody was obtained in the APC channel. The intensity was quantified by geometric mean (GM).

### In Vitro Cumulative Release and Colloidal Stability

The colloidal stability of Doxil and T‐DOX was studied at 37 °C in PBS containing 10% FBS. The particle size assessment was conducted at 0, 6, 12, 18, 24, and 30 h. In the cumulative release study, Doxil and T‐DOX were incubated in dialysis bags and extracted with 150 µL medium at 1, 4, 8, 12, 16, and 24 h for DOX measurement at ex/em 470/590 nm using a microplate reader.

### In Vitro Cellular Uptake

The cellular uptake of Doxil or T‐DOX was measured using CLSM and a FACSCalibur flow cytometer. Briefly, 1 × 10^5^ 4T1 cells were seeded in a 12‐well plate overnight. Cells were incubated with Doxil or T‐DOX in fresh medium for various periods. The nuclei were stained with DAPI before CLSM imaging. The cells were harvested and resuspended in 300 µL PBS, and the fluorescence signal was detected using a flow cytometer for quantitative evaluation.

### Cell Viability Assay

The MTT assay was used to evaluate the in vitro cytotoxicity of Doxil and T‐DOX against 4T1 cells. 4T1 cells (5 × 10^3^) were seeded in 96‐well plates overnight. Cells were then exposed to a cell culture medium containing a specific concentration of DOX solution (DOX‐Sol), Doxil, or T‐DOX for 24 and 48 h, respectively. The MTT solution was added to the plate and incubated for another 4 h. Then, 150 µL of dimethyl sulfoxide (DMSO) was added, and the absorbance was recorded at 570 nm using a microplate spectrophotometer.

### Western Blot Analysis

Protein samples were separated by 10% SDS‐PAGE and transferred onto polyvinylidene fluoride (PVDF) membranes. The PVDF membrane was blocked with 5% skim milk at 25 °C for 1 h, washed with TBST, and incubated at 4 °C with primary antibodies overnight. The membrane was washed with TBST and incubated with anti‐mouse IgG horseradish peroxidase (HRP)‐linked antibody (ABclonal Biotechnology) at 25 °C for 1 h. The expected proteins were detected using an ECL chemiluminescence detection kit (Meilun, China). GAPDH was used as an internal standard.

### Immunohistochemistry and Immunofluorescence Staining

Tumor tissues were embedded in paraffin after fixation in 10% formalin before IHC staining. Four‐µm‐thick sections were de‐waxed, rehydrated with graded alcohols, and then mounted on glass slides. Following PBST (phosphate buffered saline containing 0.05% Tween‐20) rinses, the slides were incubated with the primary antibodies for 1 h and then incubated with appropriate HRP‐conjugated secondary antibodies at room temperature for 30 min. The slides were washed with PBST, incubated with DAB (3, 3′‐diaminobenzidine), and then scanned with an Aperio AT Turbo Digital Pathology Scanner (Leica Biosystems). IF staining was performed on tumor tissue slides by fixing, permeabilizing, blocking with 1% BSA, and incubating with antibodies overnight at 4 °C. Nuclei were counterstained with DAPI before CLSM imaging. Table S1, Supporting Information, illustrates the antibodies used.

### CRT Expression and HMGB‐1 Release

4T1 cells (1 × 10^5^) were seeded in 24‐well plates overnight. The cell culture medium was refilled with cis‐platinum (CIS), DOX‐Sol, Doxil, and T‐DOX at the indicated concentrations for 24 h. For HMGB‐1 release, the supernatants were collected and analyzed using an ELISA kit (Jymbio, Colorful Gene Biological Technology Company), according to the manufacturer's instructions. CRT exposure was measured by incubation with the primary antibody for 1 h. The cells were then washed three times and incubated with FITC‐conjugated secondary antibody for 30 min, followed by nuclear staining with DAPI before CLSM imaging.

### In Vivo Vaccination Study

4T1 cells were pre‐treated with PBS, CIS (10 µM), DOX Sol, Doxil, or T‐DOX (DOX concentration of 80 µM) for 24 h. Next, 4T1 cells (1 × 10^6^) in 100 µL fresh culture medium were injected into the left flanks of BALB/c female mice (*n* = 5) on two separate occasions. After treatment, 100 µL of fresh culture medium containing live 4T1 cells (1 × 10^6^) was injected into the right flanks of the mice. Figure 3d illustrates the experimental procedure and the timeline. The tumor volume (0.5 × length × width^2^) was measured using a vernier caliper. Tumors were collected for immunofluorescence staining and cytokine evaluation on day 23 post‐euthanasia. IFN‐*γ* and TNF‐*α* levels in tumor tissues were evaluated using an ELISA kit.

### In Vivo Tumor‐Targeting Study

A random sample of 4T1 tumor‐bearing mice was divided into two groups. As the tumor volume approached 200 mm^3^, the mice were injected intravenously with either DiR‐labeled liposomes (Doxil^DiR^) or hybrid exosomes (T‐DOX^DiR^) at a dose of 1 mg kg^−1^. Fluorescence imaging was performed using an IVIS at various time intervals (4, 8, 12, 24, and 48 h) following intravenous injection.

### Preparation, Characterization, and in Vivo Therapeutic Effect of S‐PDNGs

PD1 immune nano‐clusters were prepared. NHS‐SS‐NHS was diluted in 10 µL DMSO and mixed with the PD1 inhibitor solution (PD1 Sol) at different molecular ratios for 30 min at 25 °C under magnetic agitation. Furthermore, PD1 immune nanoclusters were co‐extruded with TDEVs at 37 °C to produce S‐PDNGs. The morphology of the S‐PDNGs was observed by TEM at 80 kV. The reduction sensitivity was assessed by placing S‐PDNGs in a dialysis bag (300 kDa) containing 1, 10, and 100 mm DTT. Dialysis medium was extracted and read using a microplate reader at 280 nm at different intervals.

The in vivo therapeutic effects of S‐PDNGs were tested in 4T1 orthotopic tumor‐bearing mice. Figure 4d illustrates the experimental procedure and the timeline. When the tumor volume approached 200 mm^3^, mice were randomly assigned to three groups (*n* = 5). Each group was administered PBS, PD1 Sol, or S‐PDNGs (50 µg PD1/mouse). For safety evaluation, the tumor volume was measured accurately using a vernier caliper and body weight. After treatment, the mice were euthanized to collect whole blood and spleens for Th17 cell measurement and H&E staining.

### Sequential Dosing of T‐DOX and S‐PDNGs

The optimal administration sequence based on T‐DOX and S‐PDNGs was studied in 4T1‐luc tumor‐bearing mice. Figure 4j illustrates the experimental procedure and the timeline. Five groups of 4T1‐luc tumor‐bearing mice (*n* = 3) were randomly assigned to each group when the tumor volume reached 150 mm^3^. Groups I (1TP) and II (1PT) were administered T‐DOX and S‐PDNGs consecutively, but in the opposite order, with a 1‐day interval. Groups III (7TP) and IV (7PT) were provided with T‐DOX and S‐PDNGs sequentially, but in the opposite order, with a 7‐day interval. The final group was treated with PBS as the negative control. For bioluminescence imaging, each mouse was tail‐vein injected with 200 µL D‐fluorescein at a concentration of 15 mg mL^−1^ before visualization using an IVIS imaging system.

### In Vivo Therapeutic Efficacy Study

For orthotopic breast carcinoma studies, female BALB/c mice were inoculated with 2 × 10^5^ 4T1 cells per mouse onto the left mammary fat pad by subcutaneous injection. When the tumor volume approached 200 mm^3^, mice were randomly assigned to four groups (*n* = 5) and intravenously administered PBS, Doxil, T‐DOX, or T‐DOX + S‐PDNGs (5 mg kg^−1^ DOX, 50 µg PD1/mouse). PBS, Doxil, and T‐DOX were administered to the mice on days 0, 2, 4, 6, and 8. For combinatorial chemoimmunotherapy, S‐PDNGs were administered one day after T‐DOX treatment on days 2, 6, 9, and 11. Accurate tumor volume was measured using a vernier caliper, and body weight was measured for safety evaluation. On day 15, the mice were sacrificed and the plasma, spleen, and tumor tissues were obtained for immunological evaluation. Figure 5a illustrates the experimental procedure and the timeline.

### Flow Cytometry and Cytokine Assay for Immune Response

The tumor and spleen tissues were dissected and digested at 37 °C with collagenase IV for 1 h. The digested tissues were then strained twice using a 70 µM cell strainer to obtain single‐cell suspensions. The single cells were resuspended in staining buffer and stained with fluorescent conjugated antibodies to detect 1) CD8^+^ T cells (CD3^+^CD8^+^), 2) mature DCs (CD80^+^CD86^+^), 3) memory T cells (CD44^+^CD62L^−^), 4) Tregs (CD4^+^Foxp3^+^), and 5) Th17 cells (CD4^+^IL‐17A^+^). The experimental data were analyzed using the FlowJo V10 software (TreeStar). An ELISA kit was used to determine the levels of IFN‐*γ* and TNF‐*α* in the tumor tissues. Table S1, Supporting Information, lists the antibodies used.

### Blood Chemistry Analysis and Hematoxylin and Eosin Staining

The mice were sacrificed to obtain serum. Hepatic and renal functions were evaluated based on hematological indices, including alanine aminotransferase, aspartate aminotransferase, urea nitrogen, and creatinine. The major organs (heart, liver, spleen, lungs, and kidneys) were collected and fixed in 4% formaldehyde before H&E staining. Images of the H&E‐stained tissues were captured using a bright‐field light microscope (Olympus BX61).

### Statistical Analysis

GraphPad Prism (Version 9.0) was used for statistical analysis. Data between two groups were analyzed by independent Student's t‐test. Data among multiple groups were analyzed by one‐way analysis of variance. Significant values are presented as mean ± standard deviation (SD). The *p < 0.05, **p < 0.01, and ***p < 0.001 were considered significant.

### Ethics Approval

All research involving human samples were approved by the Institutional Review Board of the First Hospital, China Medical University (Protocol Number: [2022]221). The animal experiments were approved by the Animal Ethics Committee of China Medical University (No. CMU NKT 2022211).

## Conflict of Interest

The authors declare no conflict of interest.

## Author Contributions

M.S. and W.S. contributed equally to this work. J.S., Z.H., and Q.L. designed research; M.S., W.S., and Y.W. performed the research; M.S., W.S., and Y.W. analyzed the data; M.S. wrote the manuscript. S.C. and Q.L. participated in the manuscript revision.

## Supporting information

Supporting Information

## Data Availability

Research data are not shared.
