# Correction to “Immunogenic Nanovesicle‐Tandem‐Augmented Chemoimmunotherapy via Efficient Cancer‐Homing Delivery and Optimized Ordinal‐Interval Regime”

**DOI:** 10.1002/advs.202520147

**Published:** 2025-12-20

Mengchi Sun, Wen Shi, Yuxia Wu, Zhonggui He, Jin Sun, Shuang Cai, and Qiuhua Luo.

DOI: 10.1002/advs.202205247. Adv Sci (Weinh) 2023 January; 10(1):2205247.

In Figure 4g, the flow cytometry plot for “2.28% Th17 cells in spleen (S‐PDNG)” was accidentally overlaid with the plot for “2.20% Th17 cells in spleen (PBS)” due to a formatting error in the image layout. The corrected version of the Figure is presented below:


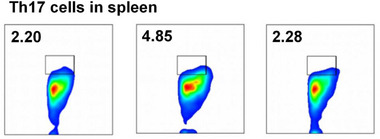


In Figure 4k, numerical labels for four data points (D7 (n1) of 1TP, D1 (n3) of 1PT, D7 (n3) of 1PT, and D22 (n1) of 7TP) were incorrectly inputted during the figure preparation process. The corrected version of the Figure is presented below:


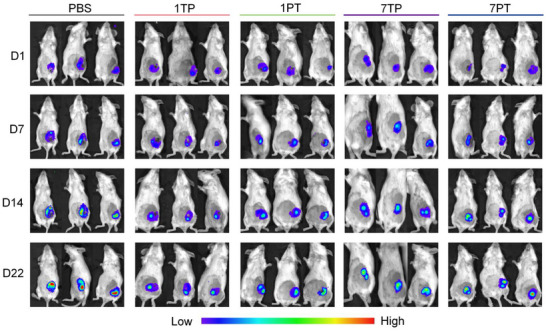


The H&E staining image of spleens in the T‐DOX group (Figure S20, Supporting Information) was accidentally overlaid with the H&E staining image of spleens in the PBS group (Figure 4i) due to a typesetting error. The corrected version of Figure S20 (Supporting Information) is presented below:


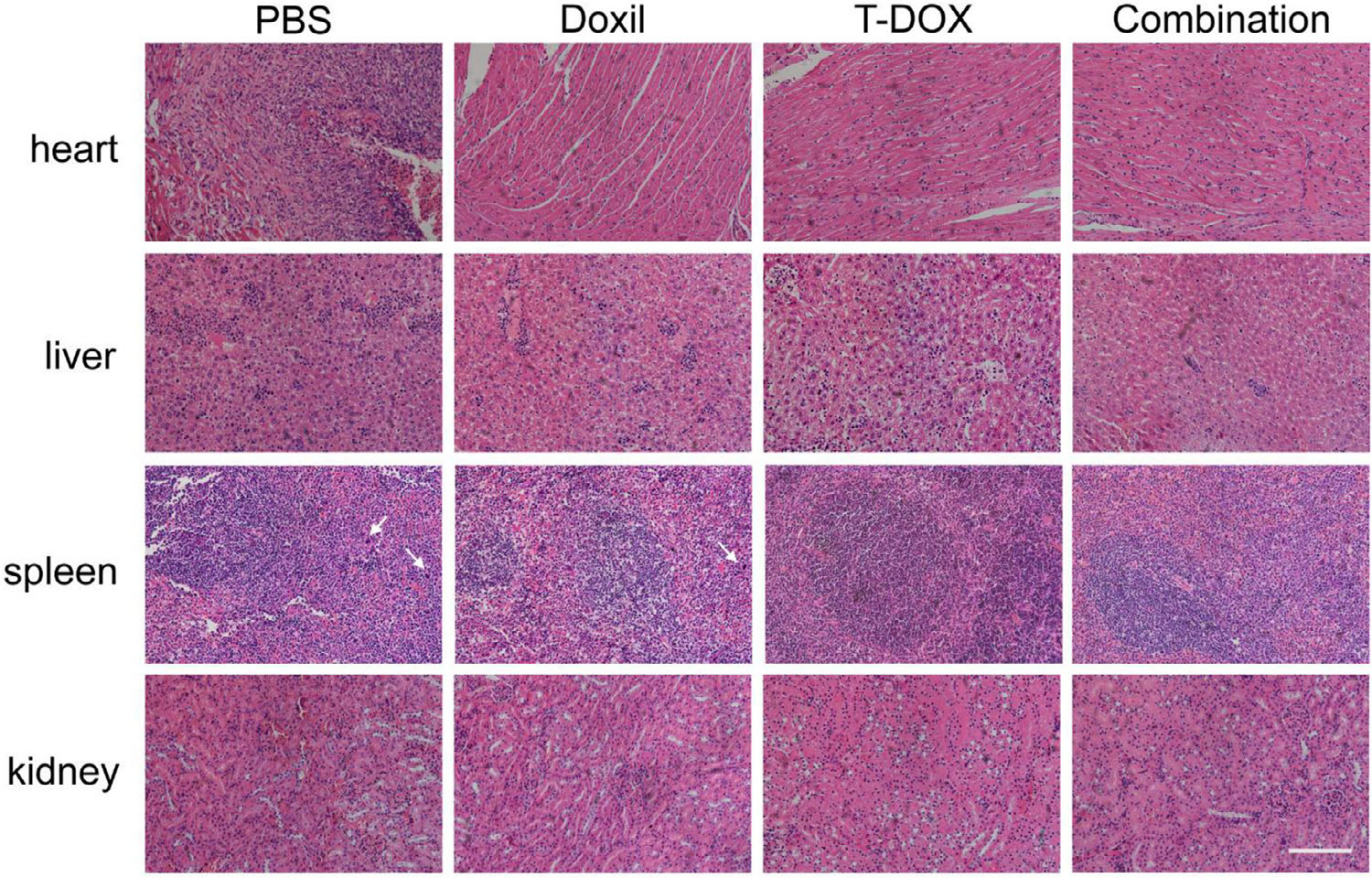


We apologize for the errors.

