# Supplementary material for: Immunogenic Nanovesicle‐Tandem‐Augmented Chemoimmunotherapy via Efficient Cancer‐Homing Delivery and Optimized Ordinal‐Interval Regime
Source: Adv Sci (Weinh). 2022 Dec 1;10(1):2205247. doi: 10.1002/advs.202205247 (PMC9811449; doi:10.1002/advs.202205247)
Supplement: Supplementary file 1 — Supporting Information [file ADVS-10-2205247-s001.pdf]

ADVANCED SCIENCE
Open Access

Supporting Information

for *Adv. Sci.*, DOI 10.1002/advs.202205247

Immunogenic Nanovesicle-Tandem-Augmented Chemoimmunotherapy via Efficient Cancer-Homing Delivery and Optimized Ordinal-Interval Regime

Mengchi Sun, Wen Shi, Yuxia Wu, Zhonggui He, Jin Sun, Shuang Cai and Qiuhua Luo**

Supporting Information

Immunogenic Nanovesicle-Tandem-Augmented Chemoimmunotherapy *via* Efficient Cancer-Homing Delivery and Optimized Ordinal-Interval Regime

Mengchi Sun#, Wen Shi#, Yuxia Wu, Zhonggui He, Jin Sun, Shuang Cai, Qiuhua Luo**

W. Shi, Y. Wu

Department of Pharmacy, The First Hospital of China Medical University, Shenyang, Liaoning, 110001, P. R. China

M. Sun, Z. He, J.Sun

Wuya College of Innovation, Shenyang Pharmaceutical University, Shenyang, Liaoning, 110016, P. R. China

#These authors contributed equally to this work.

*Corresponding author, Q.L, S.C

Department of Pharmacy, The First Hospital of China Medical University, Shenyang, Liaoning, 110001, P. R. China

E-mail: qhluo@cmu.edu.cn; caishuang1972@126.com

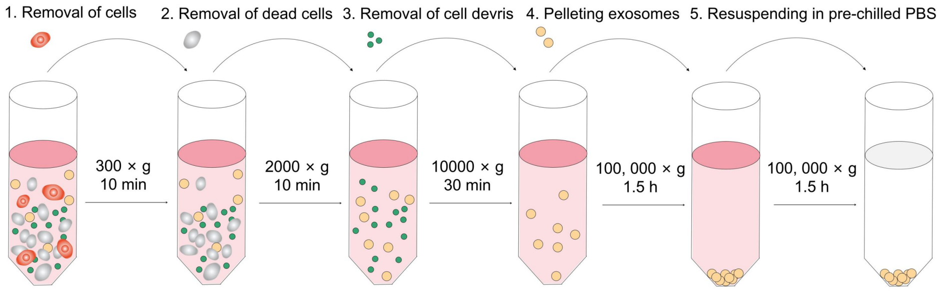

Figure S1 Schematic representation of TEDV isolation using the differential centrifugation method.

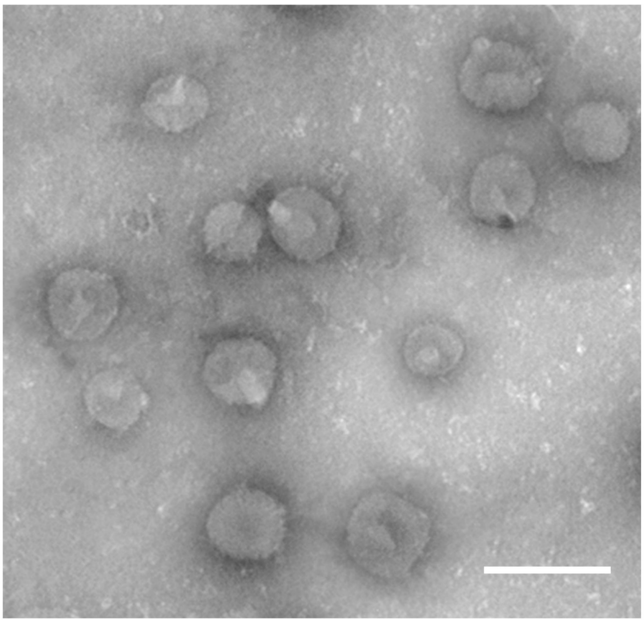

Figure S2 TEM image of TEDV. Scale bar = 200 nm.

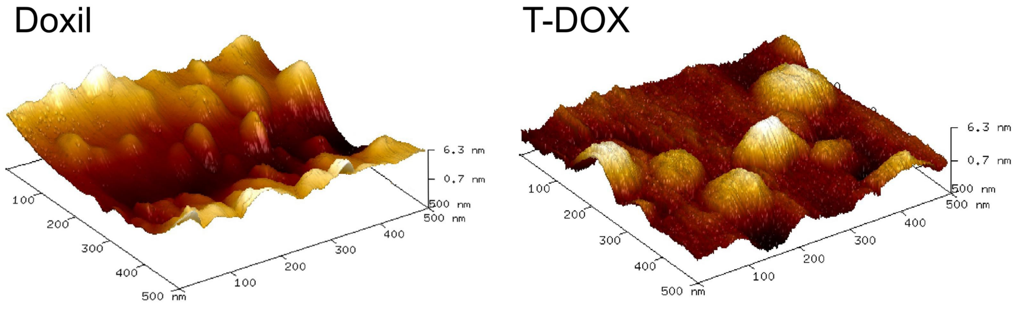

Figure S3 AFM images of Doxil and T-DOX.

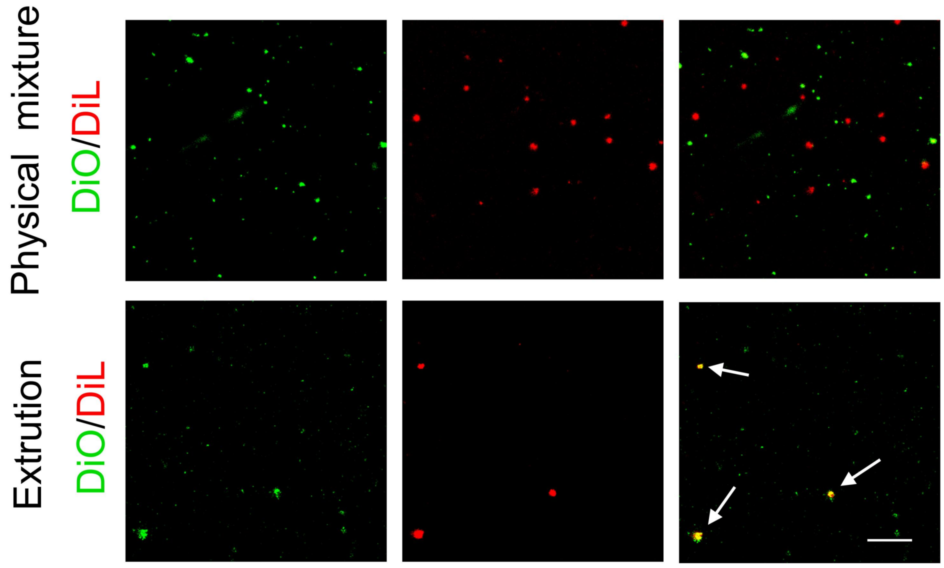

Figure S4 CLSM images of physical mixture and extrusion of TEDVs and liposomes. (red = liposome, green = TEDV; Scale bar = 2 μm).

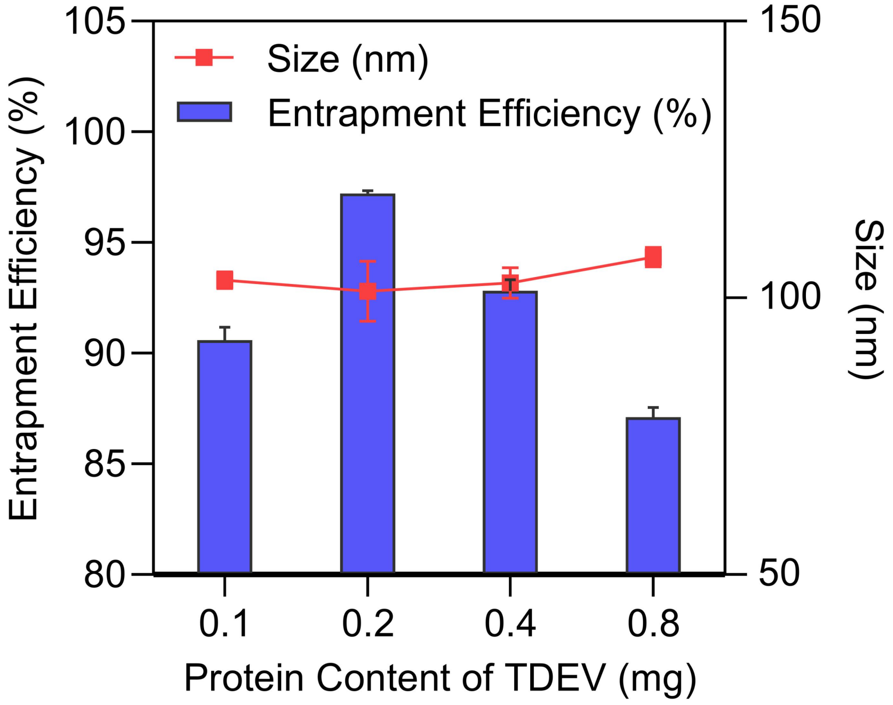

Figure S5 Hydrodynamic diameter and entrapment efficiency of T-DOX with different formulations (n = 3).

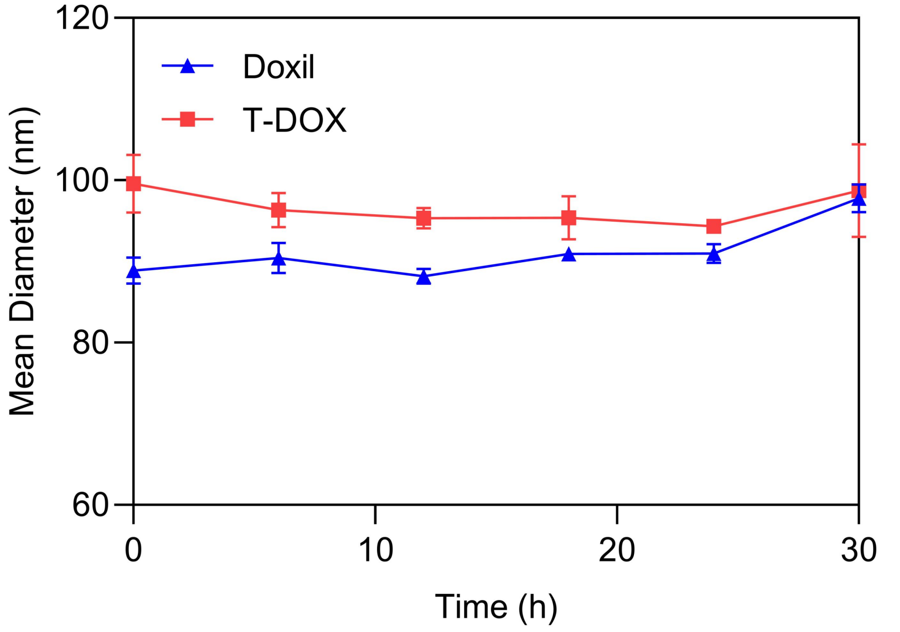

Figure S6 *In vitro* stability of Doxil and T-DOX in PBS (pH 7.4) containing 10% fetal bovine serum (FBS) for 30 h (n = 3).

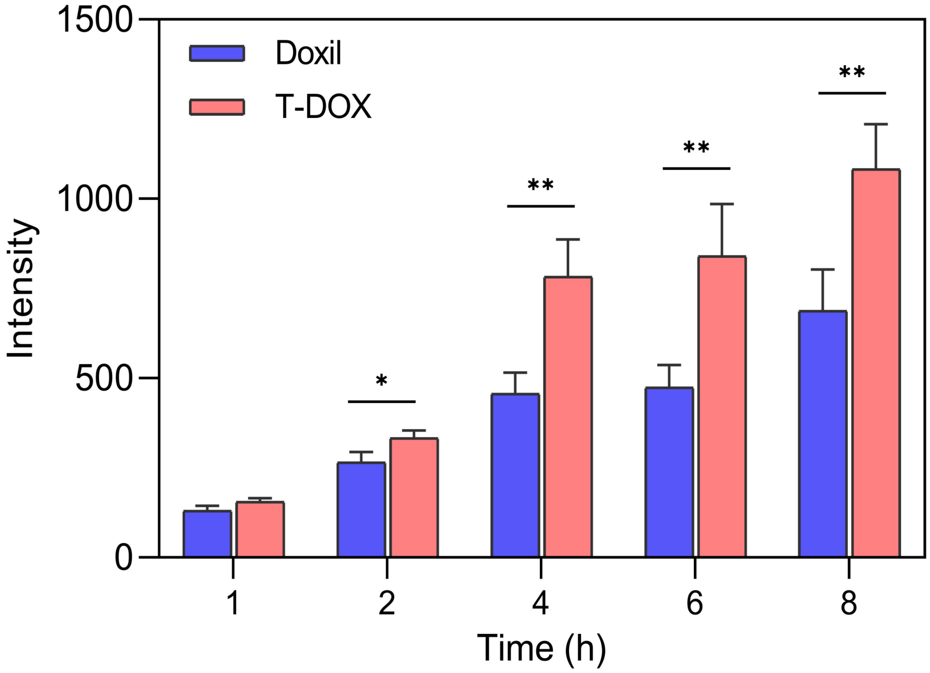

Figure S7 Flow cytometry analysis of 4T1 cells incubated with Doxil or T-DOX at different time points (n = 3). Significant values are presented as mean ± SD; $*P < 0.05$; $**P < 0.01$; $***P < 0.001$.

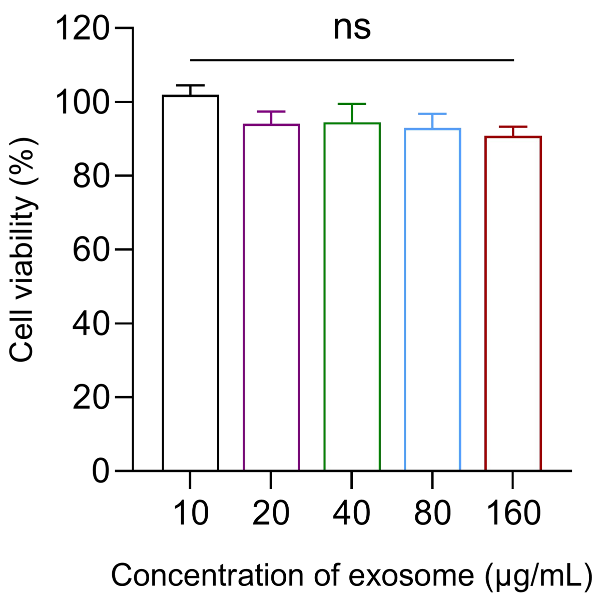

Figure S8 *In vitro* cytotoxicity of blank biohybrid exosome against 4T1 cells for 48 h (n = 3).

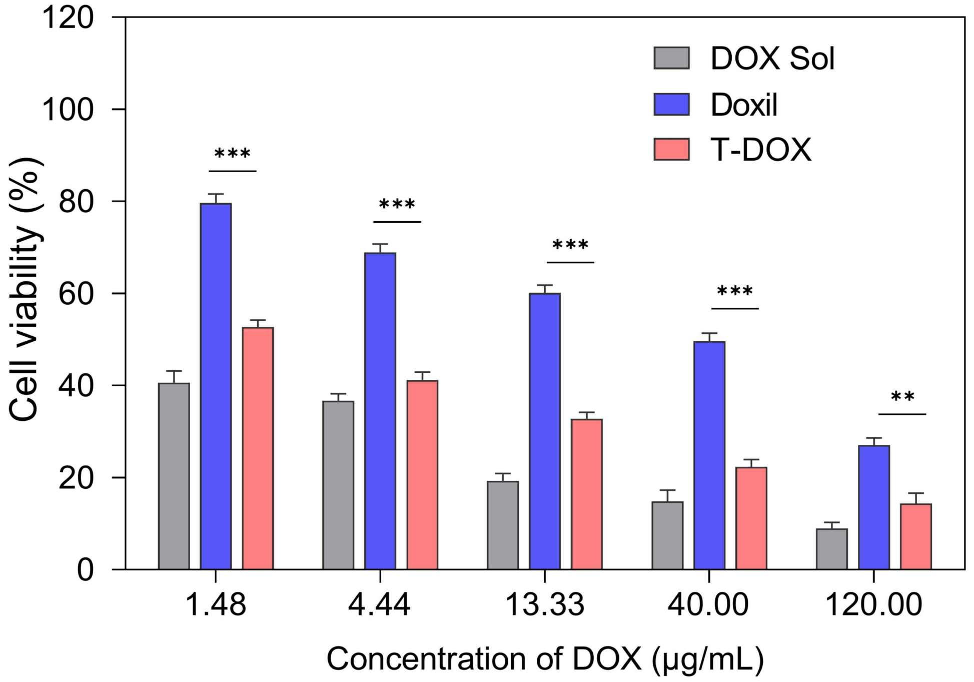

Figure S9 *In vitro* cytotoxicity of DOX Sol, Doxil, or T-DOX against 4T1 cells for 48 h (n = 3). Significant values are presented as mean ± SD; *P＜0.05; **P＜0.01; ***P＜0.001.

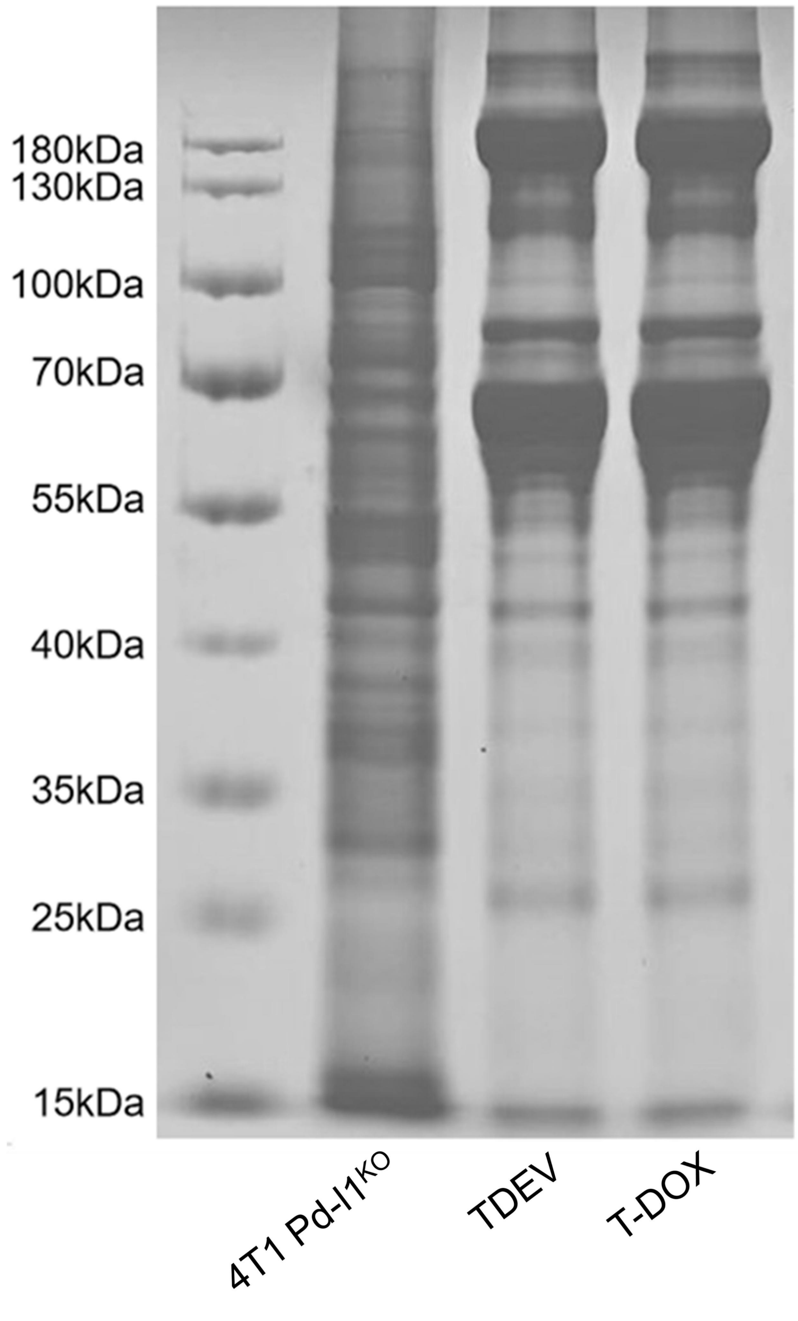

Figure S10 SDS-PAGE protein analysis of 4T1 Pd-l1^{KO}, TEDV, and T-DOX.

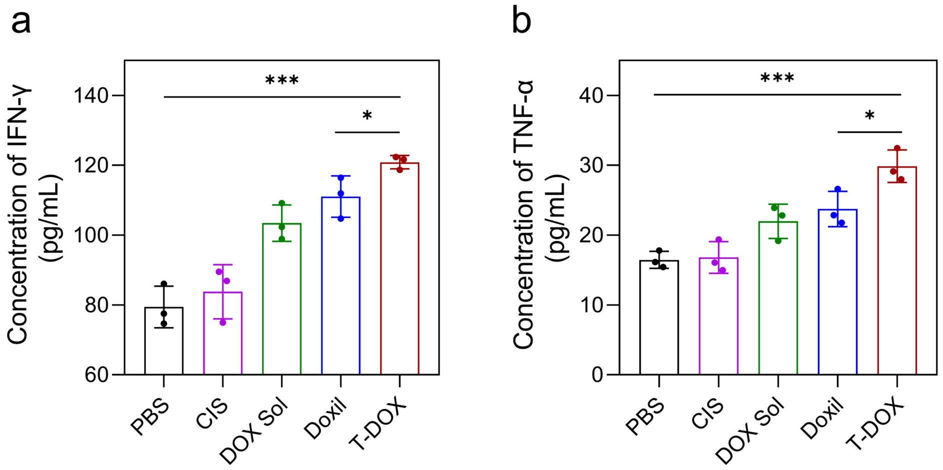

Figure S11 (a) IFN-γ and (b) TNF-α in tumors following various treatments (n = 3). Significant values are presented as mean ± SD; *P＜0.05; **P＜0.01; ***P＜0.001.

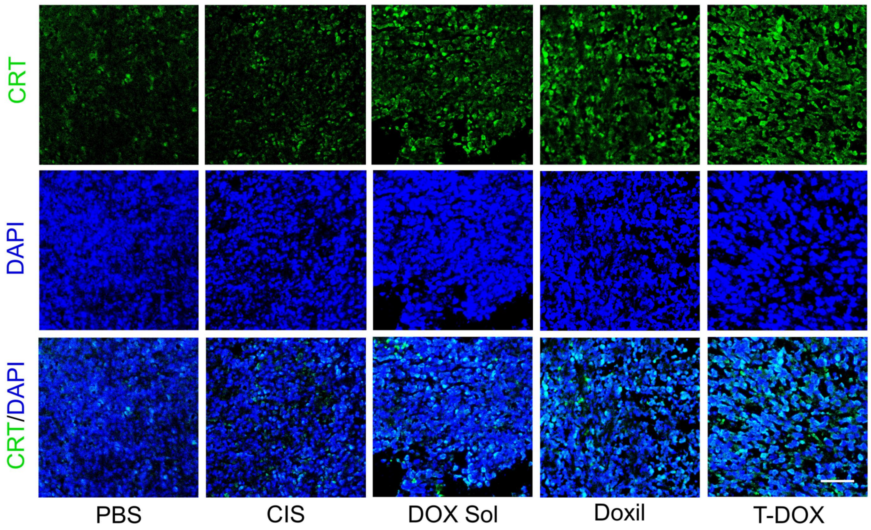

Figure S12 CRT staining of tumor slices after various treatments. Scale bar = 50 μm.

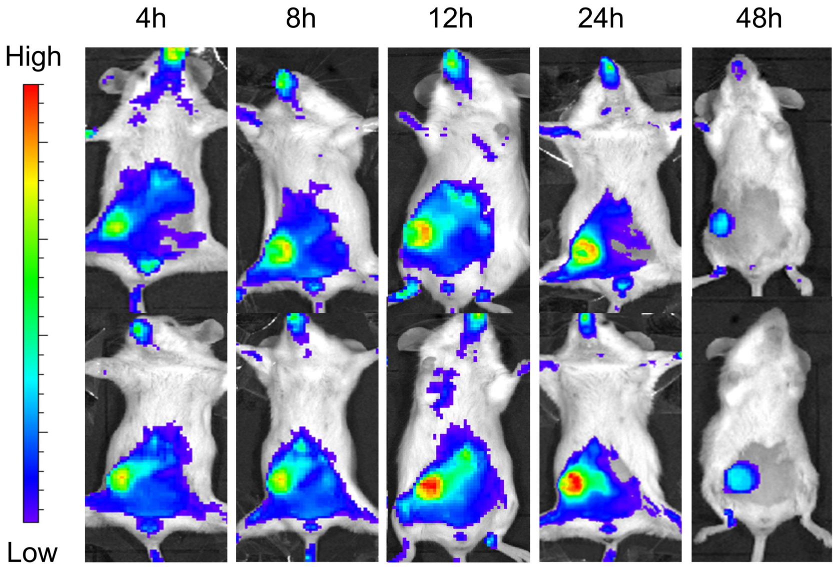

Figure S13 *In vivo* fluorescence imaging in orthotopic 4T1 tumor-bearing mice at 4 h, 8 h, 12 h, 24 h, and 48 h following injection with Doxil^{DiR} and T-DOX^{DiR}.

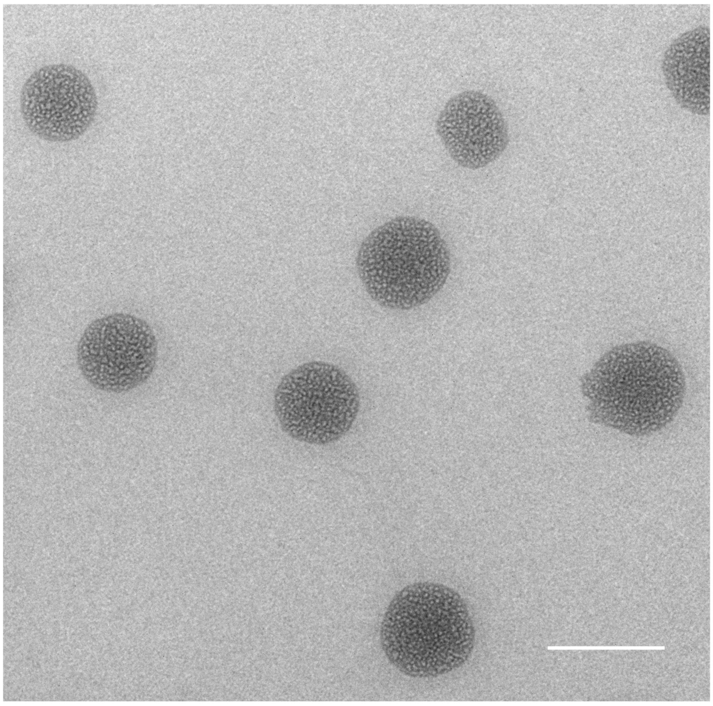

Figure S14 TEM image of S-PD. Scale bar = 200 nm.

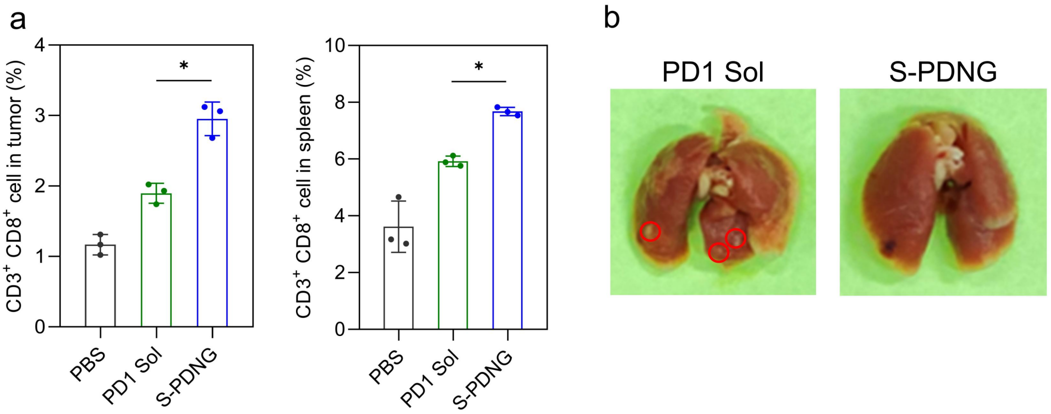

Figure S15 (a) $CD8^+$ T cell ratios in tumors and splenocytes of the 4T1 tumor-bearing mice after various treatments (n = 3). Significant values are presented as mean ± SD; *P＜0.05; **P＜0.01; ***P＜0.001. (b) Lung tissues collected from various treatments. Red circles demonstrate the visible metastatic site.

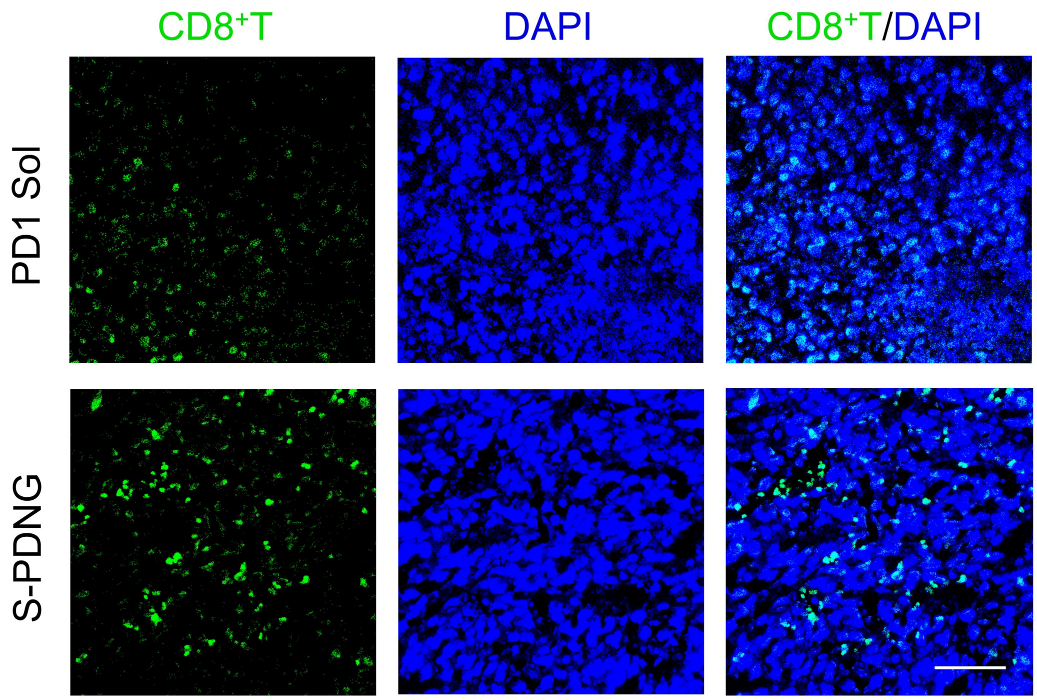

Figure S16 CD8 staining of tumors slices following PD1 Sol and S-PDNG treatment. Scale bar = 50 μm.

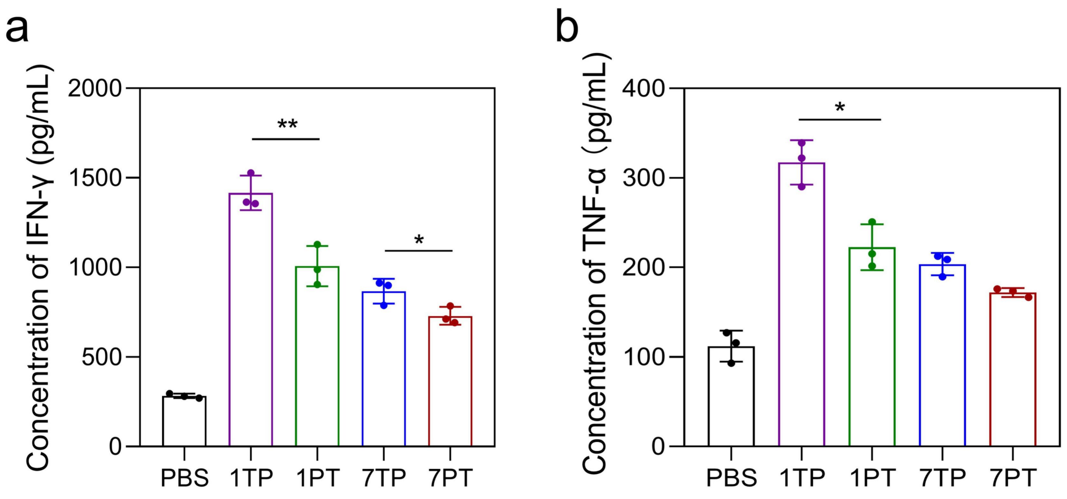

Figure S17 (a) IFN-γ and (b) TNF-α in tumors following sequential dosing of T-DOX and S-PDNGs. (n = 3). Significant values are presented as mean ± SD; *P＜0.05; **P＜0.01; ***P＜0.001.

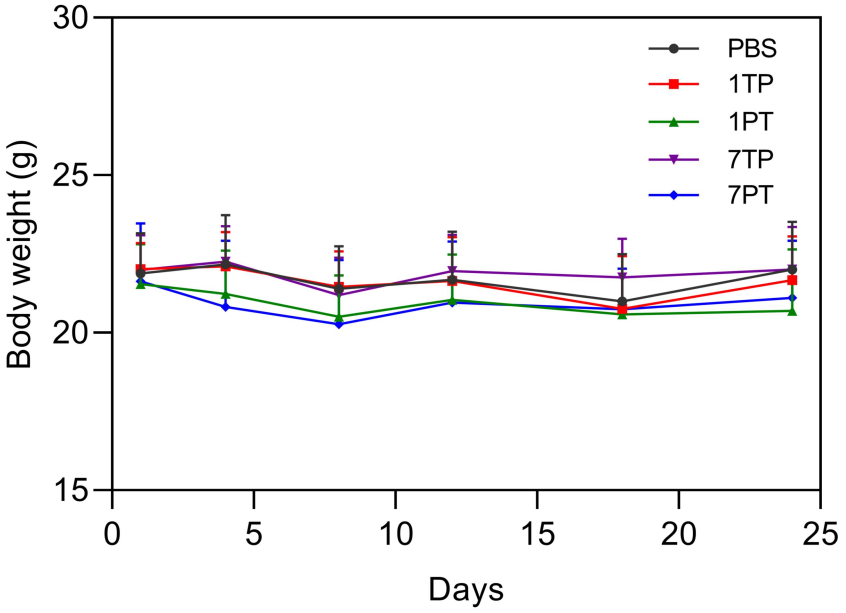

Figure S18 Body weight of mice following sequential dosing of T-DOX and S-PDNG (n = 3).

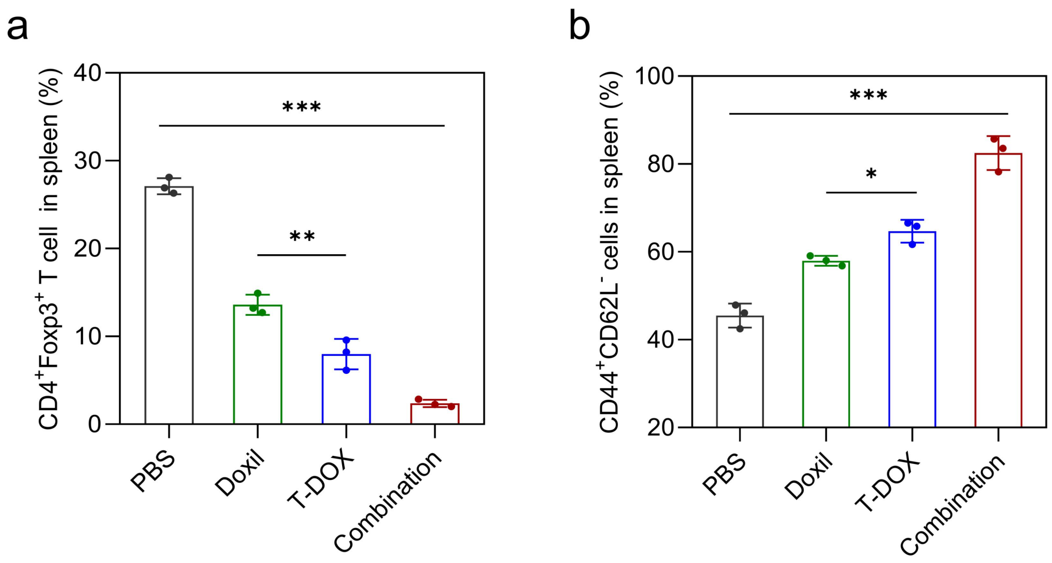

Figure S19 (a) Treg and (b) Memory T cell ratios in splenocytes of 4T1 tumor-bearing mice after various treatments (n = 3). Significant values are presented as mean ± SD; *P＜0.05; **P＜0.01; ***P＜0.001.

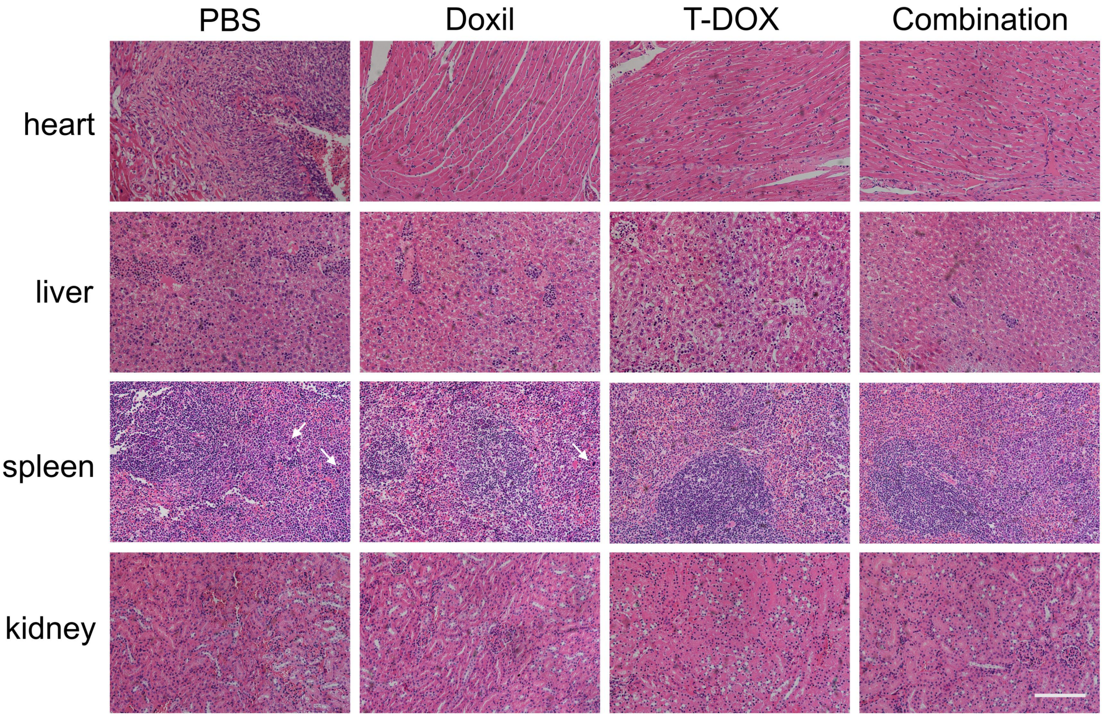

Figure S20 Pathological analyses of heart, liver, spleen, and kidney of the orthotopic 4T1 tumor-bearing mice in the PBS, Doxil, T-DOX, and combination-treated groups on day 11. White arrows demonstrate the lung metastases. Scale bar = 100 μm.

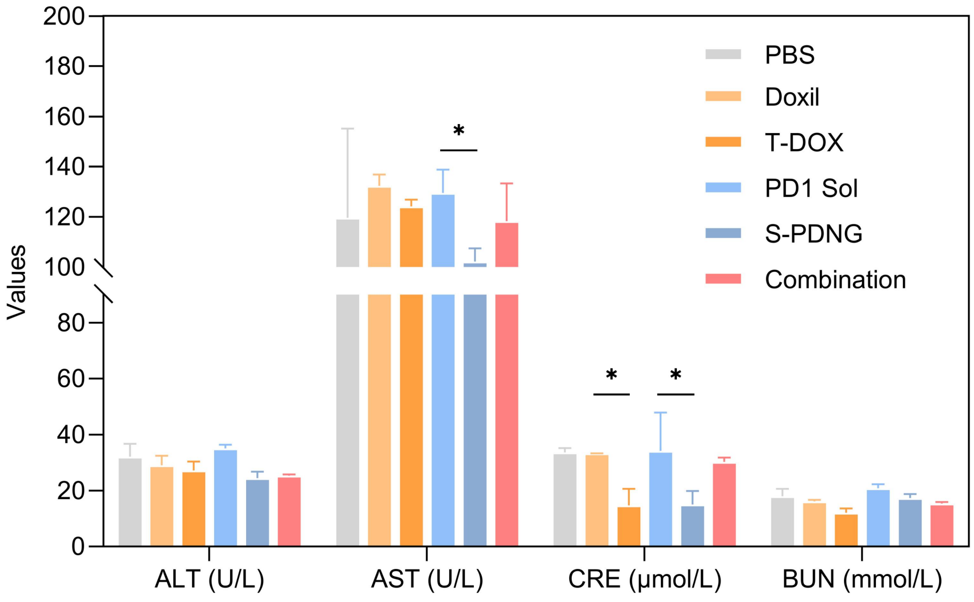

Figure S21 Blood chemistry analysis of the orthotopic 4T1 tumor-bearing mice after various treatments (n = 3). Significant values are presented as mean ± SD; *P＜0.05; **P＜0.01; ***P＜0.001.

Table S1 Antibodies used in the study.

Antibodies	Company	Catalog No.	Application	Dilution fold
Anti-CD4	ABclonal	A0362	WB, IF	500, 50
Anti-CD8a	Bioss	bs-10699R	WB, IF	1000, 200
Anti-CD81	ProteinTech	66866-1-Ig	WB	1000
Anti-TSG101	ProteinTech	67381-1-Ig	WB	5000
Anti-CRT	ProteinTech	27298-1-AP	WB, IF	2000, 250
APC Anti-PD-L1	Abcam	ab205921	FC	200
Anti-PD-L1	ProteinTech	66248-1-Ig	WB,IF	2000,50
FITC anti-CD3	Biolegend	100204	FC	200
PercP anti-CD4	Biolegend	100539	FC	200
APC anti-CD8A	Biolegend	300926	FC	200
PercP anti-CD3	Biolegend	103132	FC	200
PE anti-CD44	Biolegend	100422	FC	200
FITC anti-CD8	Biolegend	100706	FC	200
APC/Cy7 anti-CD62L	Biolegend	104428	FC	200
PE anti-CD86	Biolegend	105007	FC	200
FITC anti-CD80	Biolegend	104705	FC	200
PE anti-FoxP3	Biolegend	126403	FC	200
APC anti-IL-17a	Biolegend	506915	FC	200
